# Supplementary material for: HIV viral load non-suppression and associated factors among pregnant and postpartum women in rural northeastern South Africa: a cross-sectional survey
Source: BMJ Open. 2022 Mar 10;12(3):e058347. doi: 10.1136/bmjopen-2021-058347 (PMC8915310; doi:10.1136/bmjopen-2021-058347)
Supplement: Supplementary data [file bmjopen-2021-058347supp002.pdf]

Summary of participant characteristics by maternal viral load non-suppression per study group

	Third trimester		0-14 weeks postpartum		15-26 weeks postpartum		27-52 weeks postpartum		53-104 weeks postpartum	
	**N**	**% VL>1000 [95% CI]**	**N**	**% VL>1000 [95% CI]**	**N**	**% VL>1000 [95% CI]**	**N**	**% VL>1000 [95% CI]**	**N**	**% VL>1000 [95% CI]**
All	176	17.1 [11.7, 24.2]	128	15.0 [8.5, 25.2]	75	6.9 [2.2, 20.1]	123	17.5 [9.0, 31.2]	110	16.4 [9.3, 27.2]
**Socio-demographics and antenatal care**										
Age in years										
15–24	33	28.7 [13.2, 51.6]	23	3.1 [0.2, 28.9]	12	16.9 [1.2, 76.6]	17	28.0 [10.4, 56.5]	20	39.0 [25.9, 54.0]
25-34	99	15.4 [7.7, 28.4]	75	17.0 [6.8, 36.4]	48	6.0 [1.5, 21.5]	73	16.7 [6.3, 37.4]	56	14.5 [4.4, 38.3]
35-46	44	12.1 [3.2, 36.8]	30	20.2 [7.3, 44.9]	15	0.0	33	14.2 [3.9, 40.8]	34	5.3 [1.2, 20.6]
P-values		*0.332*		*0.288*		*0.471*		*0.538*		***0.016***
BMI										
13.0 to 24.9	33	12.1 [3.2, 36.4]	48	19.1 [5.7, 47.8]	33	12.7 [4.2, 32.6]	53	12.8 [6.2, 24.4]	53	19.0 [8.1, 38.6]
25 to 29.9	55	25.9 [15.1, 40.7]	41	20.8 [6.4, 50.3]	19	4.3 [0.4, 33.2]	39	25.2 [7.0, 60.3]	21	23.3 [12.2, 39.9]
30 to 76.7	86	13.9 [7.9, 23.2]	39	2.1 [0.2, 19.3]	22	0.0	30	16.7 [7.5, 33.2]	35	9.0 [3.4, 21.4]
P-values		*0.175*		*0.274*		*0.115*		*0.351*		*0.184*
Education										
>12 years of education	59	17.9 [6.3, 41.6]	59	13.1 [6.7, 24.0]	23	3.9 [0.6, 21.3]	43	22.1 [9.4, 43.7]	29	8.6 [1.3, 40.3]
≤12 years of education	117	16.7 [9.7, 27.2]	69	16.4 [6.9, 34.1]	52	8.1 [2.4, 24.0]	80	14.9 [8.8, 23.9]	81	19.3 [10.6, 32.4]
P-values		*0.899*		*0.644*		*0.335*		*0.192*		*0.324*
Married/Cohabiting										
No	114	15.3 [8.0, 27.3]	77	8.2 [4.0, 16.2]	46	1.9 [0.2, 17.2]	68	18.5 [11.1, 29.2]	57	16.1 [7.8, 30.2]

Yes	62	20.7 [11.1, 35.2]	51	24.5 [9.5, 49.9]	29	13.1 [4.7, 31.6]	55	16.4 [3.8, 49.0]	53	16.6 [7.9, 31.6]
P-values		*0.480*		*0.083*		***0.045***		*0.846*		*0.929*
Income source[a]										
Employed	47	13.4 [6.1, 27.1]	41	8.6 [2.3, 27.4]	22	0.0	26	11.6 [2.7, 37.9]	27	22.0 [5.0, 60.3]
Spouse/partner	54	20.5 [9.3, 39.1]	37	24.3 [6.3, 60.5]	19	19.0 [6.5, 44.0]	40	18.8 [4.5, 53.5]	37	15.4 [6.9, 30.6]
Parent/relative	34	24.2 [7.0, 57.4]	17	30.1 [9.5, 63.8]	9	10.3 [0.6, 69.5]	14	24.4 [8.1, 54.2]	16	26.7 [6.8, 64.5]
Grant	38	12.7 [3.9, 33.8]	32	4.7 [0.9, 20.2]	24	0.0	43	17.3 [7.0, 36.8]	29	9.0 [1.7, 35.9]
P-values		*0.580*		*0.135*		*0.146*		*0.754*		*0.513*
Household gross income/month										
>R3200	72	21.7 [15.4, 29.7]	60	15.1 [6.0, 33.1]	34	5.2 [1.4, 18.0]	55	27.2 [14.6, 45.0]	41	18.7 [6.3, 43.9]
R3200 or less/none	104	13.4 [6.8, 24.9]	68	14.9 [7.6, 27.2]	41	8.1 [1.5, 33.1]	68	9.8 [5.6, 16.4]	68	15.5 [8.3, 27.0]
P-values		*0.168*		*0.983*		*0.642*		***0.007***		*0.711*
Partner's HIV status[a]										
Negative	30	24.1 [13.1, 40.1]	17	3.7 [0.2, 37.5]	15	0.0	28	11.4 [4.4, 26.5]	20	5.1 [0.4, 41.2]
Positive	71	11.8 [3.3, 34.2]	53	18.1 [7.8, 36.8]	38	14.7 [5.1, 35.5]	60	17.7 [9.3, 31.0]	57	12.5 [3.9, 33.1]
Don't know	75	19.2 [10.3, 32.9]	57	16.4 [6.3, 36.5]	22	0.0	35	21.2 [7.7, 46.6]	33	27.3 [20.2, 35.7]
P-values		*0.429*		*0.354*		***0.035***		*0.429*		*0.137*
Condom use frequency[a]										
Never	26	23.5 [12.8, 39.2]	11	11.8 [2.0, 46.4]	1	0.0	7	0.0	7	18.2 [4.2, 53.1]
Sometimes	72	17.4 [9.0, 30.9]	46	14.1 [2.4, 52.3]	24	3.1 [0.3, 23.2]	39	15.7 [8.3, 27.9]	45	6.1 [2.3, 15.3]

Always	78	15.0 [7.0, 29.4]	70	16.5 [8.1, 30.7]	49	9.3 [2.6, 28.4	75	19.7 [7.7, 42.0]	58	22.3 [13.9, 33.8]
P-values		*0.560*		*0.920*		*0.582*		*0.501*		***0.006***
Planned pregnancy										
No	99	13.1 [6.2, 25.6]	75	14.3 [4.6, 36.6]	44	8.8 [2.2, 28.8]	58	19.9 [11.6, 31.9]	54	13.5 [5.7, 28.5]
Yes	77	22.0 [14.0, 32.9]	53	16.0 [6.9, 32.9]	31	4.3 [0.7, 21.0]	65	15.3 [4.1, 43.6]	56	18.6 [9.3, 33.5]
P-values		*0.210*		*0.871*		*0.444*		*0.646*		*0.479*
Gestation at ANC-1 visit										
≤12 weeks	75	12.7 [4.6, 30.4]	86	13.1 [4.7, 31.6]	56	6.4 [1.5, 23.2]	84	19.1 [9.2, 35.5]	78	15.2 [8.0, 26.8]
13-20 weeks	66	21.1 [13.8, 30.9]	33	12.7 [3.2, 38.8]	11	13.8 [2.2, 53.6]	29	8.3 [1.6, 32.6]	26	24.8 [12.9, 42.5]
>20 weeks	35	19.7 [8.6, 39.0]	9	34.3 [15.1,60.4]	8	0.0	10	29.9 [5.2, 76.9]	6	0.0 [0.0, 0.0]
P-values		*0.442*		*0.276*		*0.418*		*0.362*		*0.201*
Number of ANC visits[a]										
0-4 visits	93	16.7 [8.3, 30.8]	34	20.9 [9.3, 40.5]	17	4.6 [0.5, 31.7]	33	14.9 [4.6, 38.6]	33	14.1 [6.9, 26.6]
5-12 visits	83	17.5 [12.6, 23.7]	93	12.8 [7.2, 21.6]	58	7.6 [2.1, 23.7]	90	18.5 [9.4, 33.2]	77	17.5 [8.8, 31.6]
P-values		*0.893*		*0.139*		*0.646*		*0.653*		*0.550*
**Primary exposure variables**										
Timing of HIV-positive result										
Before pregnancy	128	14.2 [7.4, 25.6]	81	11.1 [5.6, 20.8]	59	7.8 [2.6, 21.5]	95	15.6 [5.9, 35.2]	85	14.0 [6.3, 28.4]
At ANC-1 or after	48	24.8 [17.2, 34.3]	47	22.0 [7.0, 51.3]	16	4.3 [0.3, 43.4]	28	23.8 [8.3, 52.1]	25	23.7 [11.9, 41.5]
P-values		*0.108*		*0.296*		*0.607*		*0.523*		*0.231*
Time since ART initiation										
>12 months	97	9.8	75	9.6	61	7.5	102	15.9	107	15.9

		[5.5, 16.8]		[5.5, 16.2]		[2.4, 21.0]		[6.5, 33.9]		[8.7, 27.4]
≤12 months	79	25.8 [19.9, 32.7]	53	22.7 [9.7, 44.6]	14	4.9 [0.3, 47.6]	*21*	24.8 [6.7, 60.2]	3	34.2 [8.5, 74.3]
P-values		***0.001***		*0.093*		*0.715*		*0.539*		*0.196*
Current ART regimen[a]										
2nd/3rd line or unknown	28	21.2 [9.3, 41.5]	29	5.4 [1.1, 22.7]	14	4.8 [0.4, 36.1]	26	3.4 [0.2, 37.3]	22	8.6 [1.9, 31.2]
First line	147	16.5 [11.0, 24.0]	99	17.6 [9.9, 29.2]	61	7.5 [1.9, 25.6]	97	21.6 [12.6, 34.3]	87	18.3 [10.1, 30.8]
P-values		*0.479*		*0.136*		*0.717*		***0.048***		*0.251*
Missed an ART dose last 7 days										
No	168	16.7 [11.3, 24.0]	123	12.2 [8.0, 18.0]	72	7.2 [2.3, 20.5]	116	18.6 [9.8, 32.4]	103	16.3 [9.0, 27.5]
Yes	8	26.3 [5.3, 69.3]	5	67.1 [8.3, 97.9]	3	0.0	7	0.0	7	18.2 [4.2, 53.1]
P-values		*0.455*		***0.029***		*0.545*		*0.066*		*0.850*
Facing any ART adherence challenges										
No	121	12.6 [7.2, 21.2]	60	17.3 [7.9, 33.7]	43	6.4 [0.9, 33.5]	72	14.7 [8.1, 25.1]	80	16.7 [8.3, 30.6]
Yes	55	25.0 [13.9, 40.8]	68	12.2 [5.4, 25.1]	32	7.7 [1.9, 26.9]	51	21.1 [5.3, 56.1]	30	15.5 [4.6, 41.1]
P-values		*0.078*		*0.503*		*0.863*		*0.616*		*0.903*
**Infant related factors** (postpartum **sample only)**										
All			*127*	*15.0 [8.5, 25.2]*	*75*	*6.9 [2.2, 20.1]*	*123*	*17.5 [9.0, 31.2]*	*110*	*16.4 [9.3, 27.2]*
Infant ever breastfed										
No			49	25.1 [8.7, 54.1]	21	3.0 [0.4, 21.4]	42	22.2 [9.3, 44.1]	34	14.3 [5.4, 32.7]
Yes			79	8.3 [3.7, 17.6]	53	8.3 [2.4, 25.3]	81	15.3 [7.9, 27.6]	76	17.2 [9.0, 30.3]
P-values				*0.138*		*0.355*		*0.268*		*0.668*
Infant currently breastfeeding										

Yes			60	9.4 [3.7, 21.8]	35	10.2 [3.2, 28.1]	48	14.4 [5.7, 32.0]	20	19.6 [9.3, 36.7]
No			19	5.3 [0.7, 32.1]	18	4.3 [0.3, 39.7]	33	16.5 [9.4, 27.4]	56	16.3 [6.9, 33.8]
No response			49	25.1 [8.7, 54.1]	22	2.8 [0.3, 20.9]	42	22.2 [9.3, 44.1]	34	14.3 [5.4, 32.7]
P-values				*0.169*		*0.398*		*0.401*		*0.797*
Infant currently on ARV prophylaxis[a]										
No			37	15.2 [6.3, 32.5]	53	4.1 [1.2, 13.3]	104	15.0 [8.0, 26.2]	99	16.2 [8.2, 29.4]
Yes			90	15.1 [8.1, 26.5]	22	12.8 [3.1, 40.4]	18	26.1 [8.5, 57.5]	10	23.6 [4.0, 69.4]
P-values				*0.984*		*0.161*		*0.145*		*0.627*
Infant HIV status at enrolment										
Negative			74	17.5 [8.9, 31.6]	61	8.6 [2.7, 24.3]	113	19.0 [10.1, 33.0]	97	13.3 [6.3, 25.9]
Positive			3	0.0	1	0.0	1	0.0	5	23.1 [3.0, 74.7]
Unknown			51	12.2 [5.8, 24.1]	13	0.0	9	0.0	8	37.3 [15.9, 65.3]
P-values				*0.473*		*0.564*		*0.330*		*0.930*
Gestational age at birth[a]										
≤37 weeks			17	20.6 [12.0, 33.1]	7	0.0	20	3.0 [0.3, 27.8]	23	31.1 [21.4, 42.8]
38 - 42 weeks			110	11.9 [7.3, 19.0]	67	8.4 [2.4, 25.5]	103	20.3 [10.2, 36.3]	87	10.9 [3.9, 26.8]
P-values				*0.154*		*0.510*		*0.078*		*0.086*
Infant birth weight[a]										
Birth weight≥2.5kg			116	14.0 [7.2, 25.4]	71	6.4 [1.7, 21.0]	109	16.9 [10.3, 26.4]	96	15.8 [8.8, 26.9]
Low birth weight			12	26.2 [17.1,38.0]	3	16.7 [0.9, 81.9]	14	22.5 [3.7, 68.9]	14	20.3 [4.8, 56.0]
P-values				*0.062*		*0.499*		*0.59*		*0.68*

[a]denominator less than N due to missing responses; BMI – body mass index; ART antiretroviral therapy; ARV- antiretroviral; PMTCT – prevention of mother-to-child transmission of HIV; ANC – antenatal care; CI – confidence interval. P-values are from a chi-squared test. Significant p-values <0.05 are in boldface font.

Ngandu NK, *et al. BMJ Open* 2022; 12:e058347. doi: 10.1136/bmjopen-2021-058347
