# Supplementary material for: HIV viral load non-suppression and associated factors among pregnant and postpartum women in rural northeastern South Africa: a cross-sectional survey
Source: BMJ Open. 2022 Mar 10;12(3):e058347. doi: 10.1136/bmjopen-2021-058347 (PMC8915310; doi:10.1136/bmjopen-2021-058347)
Supplement: Author's manuscript [file bmjopen-2021-058347.draft_revisions.pdf]

BMJ Open is committed to open peer review. As part of this commitment we make the peer review history of every article we publish publicly available.

When an article is published we post the peer reviewers' comments and the authors' responses online. We also post the versions of the paper that were used during peer review. These are the versions that the peer review comments apply to.

The versions of the paper that follow are the versions that were submitted during the peer review process. They are not the versions of record or the final published versions. They should not be cited or distributed as the published version of this manuscript.

BMJ Open is an open access journal and the full, final, typeset and author-corrected version of record of the manuscript is available on our site with no access controls, subscription charges or pay-per-view fees (http://bmjopen.bmj.com).

If you have any questions on BMJ Open's open peer review process please email info.bmjopen@bmj.com

BMJ Open

HIV viral load non-suppression and associated factors among pregnant and postpartum women in rural northeastern South Africa: a cross-sectional survey

Journal:	*BMJ Open*
Manuscript ID	bmjopen-2021-058347
Article Type:	Original research
Date Submitted by the Author:	14-Oct-2021

Complete List of Authors:	Ngandu, Nobubelo; South African Medical Research Council<br>Lombard, Carl; University of Stellenbosch, Division of Epidemiology and Biostatistics<br>Mbira, Thandiwe ; South African Medical Research Council<br>Puren, Adrian; National Institute for Communicable Diseases; National Institute for Communicable Diseases<br>Waitt, Catriona; University of Liverpool Faculty of Health and Life Sciences, Department of Pharmacology<br>Prendergast, Andrew ; Queen Mary University of London<br>Tylleskär, Thorkild; Universitetet i Bergen, Centre for International health<br>Van de Perre, Philippe; INSERM UMR 1058, Pathogenesis and control of chronic infections; Université de Montpellier,<br>Goga, Ameena; South African Medical Research Council

Keywords:	HIV & AIDS < INFECTIOUS DISEASES, Community child health < PAEDIATRICS, Epidemiology < INFECTIOUS DISEASES

SCHOLARONE™
Manuscripts

I, the Submitting Author has the right to grant and does grant on behalf of all authors of the Work (as defined in the below author licence), an exclusive licence and/or a non-exclusive licence for contributions from authors who are: i) UK Crown employees; ii) where BMJ has agreed a CC-BY licence shall apply, and/or iii) in accordance with the terms applicable for US Federal Government officers or employees acting as part of their official duties; on a worldwide, perpetual, irrevocable, royalty-free basis to BMJ Publishing Group Ltd ("BMJ") its licensees and where the relevant Journal is co-owned by BMJ to the co-owners of the Journal, to publish the Work in this journal and any other BMJ products and to exploit all rights, as set out in our licence.

The Submitting Author accepts and understands that any supply made under these terms is made by BMJ to the Submitting Author unless you are acting as an employee on behalf of your employer or a postgraduate student of an affiliated institution which is paying any applicable article publishing charge ("APC") for Open Access articles. Where the Submitting Author wishes to make the Work available on an Open Access basis (and intends to pay the relevant APC), the terms of reuse of such Open Access shall be governed by a Creative Commons licence – details of these licences and which Creative Commons licence will apply to this Work are set out in our licence referred to above.

Other than as permitted in any relevant BMJ Author's Self Archiving Policies, I confirm this Work has not been accepted for publication elsewhere, is not being considered for publication elsewhere and does not duplicate material already published. I confirm all authors consent to publication of this Work and authorise the granting of this licence.

For peer review only

HIV viral load non-suppression and associated factors among pregnant and postpartum women in rural northeastern South Africa: a cross-sectional survey

Nobubelo Kwanele Ngandu (corresponding author): HIV Prevention Research Unit, South African Medical Research Council, P. O. Box 19070, Tygerberg 7505, Cape Town, South Africa. Email: Nobubelo.Ngandu@mrc.ac.za

Carl J. Lombard: Biostatistics Unit, South African Medical Research Council, Cape Town, South Africa; Division of Epidemiology and Biostatistics, Department of Global Health, University of Stellenbosch, Cape Town, South Africa

Thandiwe Elsie Mbira: HIV Prevention Research Unit, South African Medical Research Council, Cape Town, South Africa

Adrian Puren: Centre for HIV and STI, National Institute of Communicable Diseases, Johannesburg, SA, South Africa.

Catriona Waitt: Department of Pharmacology, University of Liverpool, United Kingdom; Infectious Diseases Institute, Makerere University College of Health Sciences, Kampala, Uganda

Andrew J Prendergast: Blizard Institute, Queen Mary University of London, United Kingdom; Zvitambo Institute for Maternal and Child Health Research, Harare, Zimbabwe

Thorkild Tylleskar: Centre for International Health, University of Bergen, Norway

Philippe Van de Perre: Pathogenesis and control of chronic and emerging infections, University of Montpellier, INSERM, France; Etablissement Français du Sang, Antilles University, France; CHU Montpellier, Montpellier, France

Ameena Ebrahim Goga: HIV Prevention Research Unit, South African Medical Research Council, Cape Town, South Africa; Department of Pediatrics and Child Health, University of Pretoria, Pretoria, South Africa.

Word count = 4087

For peer review only - http://bmjopen.bmj.com/site/about/guidelines.xhtml

Abstract

Objectives: We measured the prevalence of maternal HIV viral load (mVL) non-suppression and associated factors, to assess the population risk of vertical HIV transmission.

Design: Cross-sectional study

Setting: The 8 largest community health centers of Ehlanzeni, a rural district in northeast South Africa.

Participants: HIV-infected pregnant women in their third trimester and HIV-infected postpartum mothers and their biological infants, enrolled equally across all stages of the first 24 months postpartum, were included. A sample of 612 mothers was enrolled from a target of 1000.

Primary outcome measures: The primary outcome was mVL non-suppression (defined here as mVL >1000 copies/mL). We collected information on antiretroviral use, healthcare visits and socio-demographics through interviews and measured plasma mVL. Descriptive statistics, chi-square tests and multivariable logistic regression analysis were conducted.

Results: All mothers (median age 30 years) were on antiretroviral therapy (ART) and 24.9% were on ART ≤12 months. The prevalence of mVL non-suppression was 14.7% (95% confidence interval (CI) 11.3%-19.0%), while 13.8% had low-level viraemia (50-1000 copies/mL). Most (68.9%) women had initiated breastfeeding and 37.6% were currently breastfeeding their infants. Being on first-line ART (adjusted odds ratio (AOR) 2.3[95%CI 1.1-4.6]) and married/cohabiting (AOR 2.0[95%CI 1.1-3.6]) were significantly associated with increased odds of mVL non-suppression. Being older than 24 years (AOR 0.4[95%CI 0.2-0.9]) was associated with reduced odds of mVL non-suppression.

Conclusions: The prevalence of mVL ≤1000 copies/mL of 85.3% among pregnant and postpartum women living with HIV and attending public healthcare centers in this rural district is below the 2020 90-90-90 and 2030 95-95-95 UNAIDS targets. Given that low-level viraemia may also increase the risk of vertical HIV transmission, we recommend strengthened implementation of improved schedules for monitoring mVL and ART and intensified psychosocial support for younger women, while exploring district-level complementary interventions, to sustain viral loads below 50 copies/mL amongst all women.

Key words: Viral load non-suppression, pregnant, postpartum, vertical HIV transmission

For peer review only - http://bmjopen.bmj.com/site/about/guidelines.xhtml

Strengths and limitations

- This study provides mVL non-suppression data from a rural setting where research of this kind is limited.
- The mVL measurements were conducted using the gold standard whole blood plasma rather than dried blood spots despite the challenging remote settings.
- The postpartum inclusion criterion targeted mothers attending clinics with their biological children and hence could present a sample biased towards biological mothers with better clinic attendance and less representative of mothers who mostly assign child healthcare visits to other caregivers
- The sample sizes achieved were lower than planned and much lower for the postpartum stages which did not overlap with routine child vaccination schedules, but there was no statistically significant difference in the proportion of mVL non-suppression between the postpartum stages.

For peer review only

For peer review only - http://bmjopen.bmj.com/site/about/guidelines.xhtml

Background

Maternal HIV viral load (VL) during pregnancy and breastfeeding is an indicator of the risk of vertical transmission of HIV (MTCT). The HIV test-and-treat approach and universal coverage of daily life-long triple antiretroviral therapy (ART) to achieve viral suppression and prevent MTCT have contributed to substantial decreases in MTCT globally, although the incidence of vertical transmission remains high[1]. In South Africa, the national average MTCT rate has reduced to less than 2% at birth, but heterogeneity exists at sub-regional levels with much higher intrauterine infections in some districts[2]. In addition, the MTCT risk during the breastfeeding period is critical, because breast milk transmission globally is declining at a slower rate than in utero and intrapartum transmission[3]. Postpartum transmission is perpetuated by incident maternal HIV infection during the breastfeeding period combined with low use of repeat HIV testing amongst previously uninfected mothers; inadequate monitoring of maternal VL (mVL) and poor postpartum ART adherence[4-12]. Antenatal HIV prevalence has remained around 30% over the past decade in South Africa, and as high as 45% in some districts, making monitoring of mVL a necessity to ensure corrective measures in order to reduce MTCT risk[13].

In South Africa, although the target of ensuring MTCT below 5% percent at the end of breastfeeding was achieved by 2015, the case rate of less than 50 new HIV infections per 100 000 live births, to meet global elimination of MTCT (eMTCT) targets, has not been met[4 14]. Until the UNAIDS 95-95-95 goals are achieved and sustained across the PMTCT cascade from pregnancy through to the end of breastfeeding, meeting the eMTCT targets could be impossible[15 16]. The first two 95s, i.e., 95% of HIV-infected persons knowing their HIV status and 95% of these initiated on ART, are measured during the first antenatal care (ANC) visit and have been periodically reviewed to guide service delivery strengthening in South Africa[13 17]. In South Africa the UNAIDS 2020 targets of 90% are achieved for these two indicators at ANC entry level but, similar to other low- and middle-income countries (LMICs) sustaining adequate coverage of HIV diagnoses and ART adherence throughout the antenatal and postnatal period is challenging[15 18-20]. Considering that up to a third of HIV-infected pregnant women only initiate ART after enrolling into ANC, strengthening retention in care to support ART adherence and to monitor VL throughout the PMTCT cascade is critical[13]. Monitoring mVL needs to be a priority as it is the best indicator of ART efficacy, ART adherence, and need for enhanced infant postnatal prophylaxis in infants at high risk of HIV acquisition. Despite its importance, the last 95 (95% of all HIV-infected persons on ART should be virally suppressed, i.e., have VL below 1000 copies/mL) is not being monitored at a population level along the PMTCT cascade

For peer review only - http://bmjopen.bmj.com/site/about/guidelines.xhtml

in many LMICs with high HIV burden, including South Africa. This is largely attributed to the financial cost and logistical complications associated with VL measurements. Given the high heterogeneity at sub-regional levels, routine VL monitoring should be achievable by prioritizing hotspot districts currently bearing the highest antenatal and postnatal HIV burden and MTCT risk. We identified one of the hotspot districts with high maternal HIV prevalence and high MTCT in South Africa and conducted a cross-sectional evaluation of the prevalence of mVL non-suppression (VL>1000 copies/mL) and associated factors during peripartum and postpartum periods.

For peer review only

Methods

Study design and inclusion criteria

A facility-based cross-sectional study was conducted at the eight largest community healthcare centers (CHC) in Ehlanzeni district, Mpumalanga Province, in rural northeastern South Africa (bordering Mozambique); seven were in rural localities and one in a peri-urban locality. A district-level sample size was calculated using an assumed mVL non-suppression prevalence of 25% and a precision of 5% at a 90% confidence level[6 21]. The sampling strategy was designed to equally represent different PMTCT cascade milestones across the peripartum and postpartum stages and grouped according to the 2015-2019 PMTCT guidelines for infant antiretroviral prophylaxis and breastfeeding[22]. Therefore, the planned study design targeted equal sample sizes of N=200 (25 from each CHC) for each of five groups of HIV-infected women: third trimester of pregnancy, and four postpartum time points (0-14 weeks, 15-26 weeks, 27-52 weeks (6-11 months) and 53-104 weeks (12-24 months)). The inclusion criteria were: woman being in any of these stages, living with HIV and the baby present (for postpartum) on the day of interview. Data collection was conducted over a period of three months from mid-September to mid-December 2019 to minimize heterogeneity within the sample.

Data collection and laboratory measurements

The study was introduced to all female clinic attendees in waiting rooms and interested pregnant and postpartum women were screened for inclusion criteria after receiving their routine care. After written informed consent, the woman was interviewed to collect basic demographic and HIV-related clinical histories from recall and clinic records, and entered directly onto tablets linked to an electronic database (REDCap) securely managed at the host institution[23]. Whole blood was collected from all women for VL

For peer review only - http://bmjopen.bmj.com/site/about/guidelines.xhtml

measurement and transported to the local laboratory on ice. Plasma was separated within 4-12 hours and stored at -20 degrees Celsius until further analyses at the research laboratory. The HIV-1 viral load assays were performed using the Roche COBAS® AmpliPrep/COBAS® TaqMan® HIV-1 quantitative Test, version 2.0 (Roche Diagnostics GmbH, Mannheim, Germany), according to the manufacturer's instructions. The limit of virus detection using this assay was 20 copies/mL at minimum plasma volumes of 1-2 ml.

Independent variables

Sociodemographic factors: Maternal age and body mass index (BMI) were presented as categorical variables. A wide BMI range was observed and grouped into- underweight (13.0-18.4), normal (18.5-24.9), overweight (25.0-29.9), obese (30.0-39.9) and extremely obese (40.0-80.0), with extreme outliers treated as missing. Primary source of income was categorical and average household income was binary defined using the national poverty line household income cutoff of R3200/month. Marital status was either married/cohabiting or other (single, divorced, widow, undisclosed). The highest education achieved was either none, first 7 years of basic education (primary), years 8-12 (secondary), a post-secondary certificate (Tertiary-certificate) and post-secondary diploma or higher qualification (Tertiary- Diploma or higher). Participant self-reported partner's HIV status was positive, negative or unknown and frequency of condom use was never, sometimes or always.

ANC variables: Planned pregnancy (yes/no) was self-reported and gestation age at first ANC visit (≤12 weeks, 13-20 weeks, >20 weeks) and number of ANC visits (0-4 or 5-12 visits) were confirmed using clinic records for the most recent pregnancy.

ART-related factors: These included: duration on ART (≤12 months versus >12 months), current ART regimen (first-line regimen versus all others), adherence to ART defined as missed an ART dose during the most recent 7 days (yes/no), ever face challenges with ART adherence (yes/no), and timing of HIV-positive diagnosis (before most recent pregnancy versus during ANC/after).

Infant-related postnatal factors: Infant age was used to design the study strata. Child health booklets and maternal report were used to obtain infant HIV status at enrolment (positive, negative, unknown), gestational age at birth (≤37 weeks or 38-42 weeks) and birth weight (low birth weight (<2.5kg) versus higher). Mothers self-reported whether infant was currently breastfeeding (yes/no), ever breastfed (yes/no, no response) and currently on anti-HIV prophylaxis (yes/no).

Statistical analyses

For peer review only - http://bmjopen.bmj.com/site/about/guidelines.xhtml

The primary outcome measure was mVL non-suppression (VL>1000 copies/mL), in line with the South African PMTCT guideline criteria for ART regimen management at the time of data collection[22]. We conducted univariable and multivariable logistic regression analyses to measure the association between the outcome and exposures of interest. Exposure variables were included in the multivariable model only if they had an overall univariable p-value <0.2. A significance level of 0.05 in the multivariable analysis was used to indicate a significant association with mVL. Infant-related PMTCT postnatal factors were evaluated for association with mVL separately. Chi-square tests were used to assess associations between categorical variables and descriptive statistics were done. All proportions and odds ratios were adjusted (weighted) for sample size attained by multiplying by the inverse of sample-size realization proportion (proportions calculated using the facility-level sample target of N=25 within each study group stratum), and for study group stratification by specifying the strata in the survey structure. Study group stratification was not applied in the supplementary descriptive analyses of each study group separately.

The STROBE checklist for observational cross-sectional studies was followed (**Supplementary File1**).

Ethics approval was granted by the South African Medical Research Council ethics committee (EC002-2-2019) and the Regional Committee for Medical and Health Research Ethics West, Norway (REK-Vest no 2019/773).

Patient and public involvement: No patient involved.

Results

During the 3-month data collection period, interview data were successfully collected from 667 HIV infected women, translating to sample size realization of: 66% overall – within group ascertainment was 88% for third trimester group (n=176), 64% for 0-14 weeks postpartum group (n=128), 38% for 15-26 weeks postpartum group (n=75), 62% for 27-52 weeks postpartum group (n=123) and 55% for 53-104 weeks postpartum group (n=110). Very few mother-infant pairs in the 15-26 weeks postpartum group, a period with no routine vaccination schedules, were present in the clinics. VL results were available for 612/667 (91.8%) interviewed women; 55 (8.2%) samples had insufficient plasma.

For peer review only - http://bmjopen.bmj.com/site/about/guidelines.xhtml

Description of the study population

Socio-demogaphics and ANC: The median age was 30 years (interquartile range 26-35), 8.6% and 58.1% had 0-7 years and 8-12 years of completed education, respectively, while the remaining third had some tertiary education. Most (59.6%) came from households with average earnings below the poverty income line (<R3200 per month) (**Table 1**). A quarter of the participants were employed, a third relied on government grants while the remainder depended on another individual (parent/relative/spouse/partner) as their main source of income; 43.5% women were married or cohabiting; 46.4% knew they were in a concordant HIV-positive sexual relationship while 35.4% did not know their partner's HIV status. Half of the participants had an unplanned pregnancy and around two thirds had their first ANC visit during the first twelve weeks of gestation and achieved at least 5 ANC visits.

ART-related factors: Over 70% of participants were diagnosed with HIV before the current/recent pregnancy. All 612 women were already on ART at study enrolment and three quarters had been on ART for more than 12 months. Most (80.2%) women were still on their first-line regimen. Although self-reported levels of adherence to ART during the most recent 7 days were very high (95.2%), 38.0% reported ever experiencing challenges which potentially interfere with adherence to ART. The commonly cited barriers were time inconvenience (22.8%), non-disclosure of HIV status (11.7%), ART side effects (5.4%) and lack of support from family/partner (3.2%).

Infant-related postnatal factors: Close to 70% of the infants were ever breastfed and 37.6% were currently breastfeeding. Overall, 29% infants were still taking prophylaxis, higher than the 0-14 weeks postnatal sample proportion of 23% expected to be on prophylaxis. At enrolment, 79.2% of mothers knew their infants to be HIV-negative and 2.1% to be HIV-positive while 18.7% did not know the infant's HIV status. Over 80% of infants were born full-term and with weight ≥2.5 kilograms.

Table 1: Summary of study population characteristics

	n	%
All	612	100
**Study group**		
3rd trimester	147	28.8
0-14 weeks postpartum	112	20.9
15-26 weeks postpartum	70	12.2

For peer review only - http://bmjopen.bmj.com/site/about/guidelines.xhtml

27-52 weeks postpartum	102	20.1
53-104 weeks postpartum	95	18.0
**Socio-demographics and ANC**		
Age in years		
15 – 24	105	17.8
25 - 34	351	57.2
35 - 46	156	25.0
BMI[a]		
13.0 - 18.4	20	3.6
18.5 - 24.4	200	35.4
25.0 - 29.9	175	28.6
30.0 - 39.9	173	27.1
40.0 - 80.0	39	5.3
Education		
None	8	1.2
Primary (1-7 years)	44	7.4
Secondary (8-12 years)	347	58.1
Tertiary- certificate	155	23.3
Tertiary- Diploma or higher	58	10.0
Married/Cohabiting		
No	362	56.5
Yes	250	43.5
Income source[a]		
Employed	163	24.2
Spouse/partner	187	32.5
Parent/relative	90	14.2
Grant	166	29.1
Household gross income/month		
>R3200	262	40.4
R3200 or less/none	349	59.6
Partner's HIV status[a]		
Negative	110	18.1
Positive	279	46.4
Don't know	222	35.4
Condom use frequency[a]		
Never	52	7.2
Sometimes	226	35.8
Always	330	57.0
Planned pregnancy		
No	330	52.3
Yes	282	47.7

For peer review only

For peer review only - http://bmjopen.bmj.com/site/about/guidelines.xhtml

Gestation at ANC-1 visit		
≤12 weeks	379	65.8
13-20 weeks	165	23.9
>20 weeks	68	10.3
Number of ANC visits[a]		
0-4 visits	210	32.2
5-12 visits	401	67.8
**PMTCT exposure variables**		
Timing of HIV-positive result		
Before pregnancy	448	73.1
At ANC-1 or after	164	26.9
Time since ART initiation		
>12 months	442	85.1
≤12 months	170	24.9
Current ART regimen[a]		
First line	491	80.2
2nd/3rd line or unknown	119	19.8
Missed an ART dose last 7 days		
No	582	95.2
Yes	30	4.8
Facing any ART adherence challenges		
No	376	62.0
Yes	236	38.0
**Infant related factors (postpartum sample only)**	**436**	**100**
Infant ever breastfed[a]		
No	146	31.1
Yes	289	68.9
Infant currently breastfeeding		
Yes	163	37.6
No	126	31.0
Chose not to disclose	147	31.4
Infant currently on ARV prophylaxis[a]		
No	293	71.0
Yes	140	29.0
Infant HIV status at enrolment		
Negative	345	79.2
Positive	10	2.1
Unknown	81	18.7
Gestational age at birth[a]		
≤37 weeks	67	18.7
38 - 42 weeks	367	81.3

For peer review only - http://bmjopen.bmj.com/site/about/guidelines.xhtml

Infant birth weight[a]		
Birth weight ≥2.5kg	392	90.6
Low birth weight (<2.5 kg)	43	9.4

[a]denominator less than N due to missing responses; ANC- antenatal care; PMTCT – prevention of mother-to-child transmission of HIV; ART – antiretroviral therapy; ARV – antiretroviral; BMI – body mass index; ART - antiretroviral therapy.

Prevalence of maternal viral load non-suppression

Maternal viral load ranged between undetectable and 557 500 copies/mL. The median of detectable values was 138 copies/mL (interquartile range 35-3660 copies/mL). Overall, 14.7% [95% confidence interval (CI): 11.3-19.0] of the women had mVL non-suppression while 13.8% [95% CI: 10.2-18.4] had low-level viraemia (VL 50-1000 copies/mL) and 71.5% [95% CI: 63.7-78.2] had policy-defined undetectable (<50 copies/mL) mVL[22]. VL non-suppression by study group ranged between 6.9% and 17.5%, with all groups except the 15-26 weeks postpartum group, showing a prevalence of 15.0% or higher (**Table 2**). Although mVL non-suppression was lower for the participants in the 15-26 weeks group, there was no statistically significant difference across PMTCT stages (chi-squared p-value (p)=0.284).

Summary of study population characteristics by maternal viral load non-suppression

The prevalence of mVL non-suppression differed significantly by source of income (p=0.018), ART duration (p=0.029) and ART regimen (p=0.016) (**Table 2**). The proportion of VL>1000 copies/mL was higher amongst women who depended on a parent/relative or spouse/partner as their main source of income, were on ART for ≤12 months, or were on first-line ART.

Weak evidence of differences in the prevalence of mVL non-suppression were also observed when women were grouped by maternal age (p=0.054), BMI (p=0.075), household income level (p=0.097), knowledge of partner's HIV status (p=0.081) and timing of first HIV-positive result (p=0.086). The proportion of viral non-suppression tended to be higher amongst women who were: younger than 25 years of age, had BMI between 18.5-29.9, came from households with average income >R3200, aware that their partner was

For peer review only - http://bmjopen.bmj.com/site/about/guidelines.xhtml

HIV-positive or did not know their partner's HIV status or received the first HIV-positive diagnoses after enrolling into ANC for the most recent pregnancy.

Infant related postnatal PMTCT characteristics did not appear to distinguish women by prevalence of viral load non-suppression (**Table 2**).

The distribution of mVL non-suppression by participant charateristics within each study group separately is presented in the supplementary data (**Supplementary File2**).

Table 2: Summary of study population characteristics by maternal viral load non-suppression

		VL≤ 1000 copies/mL		VL> 1000 copies/mL		p-value
		n	% [95% CI]	n	% [95% CI]	
**All**		526	85.3 [81.6, 88.3]	86	14.7 [11.7, 18.4]	
Study group	3rd trimester	147	82.9 [76.9, 87.6]	29	17.1 [12.4, 23.1]	*0.284*
	0 - 14 weeks postpartum	112	85.0 [76.5, 90.8]	16	15.0 [9.2, 23.5]	
	15 - 26 weeks postpartum	70	93.1 [82.5, 97.4]	5	6.9 [2.6, 17.5]	
	27 - 52 weeks postpartum	102	82.5 [71.1, 90.1]	21	17.5 [10.0, 28.9]	
	53 -104 weeks postpartum	95	83.6 [74.5, 89.9]	15	16.4 [10.1, 25.2]	
**Maternal socio-demographics and ANC**						
Age in years	15 – 24	82	76.5 [65.5, 84.8]	23	23.5 [15.2, 34.5]	*0.054*
	25 - 34	305	86.2 [80.3, 90.5]	46	13.8 [9.5, 19.7]	
	35 - 46	139	89.5 [82.8, 93.8]	17	10.5 [6.2, 17.2]	
BMI	13.0 - 18.4	17	88.1 [63.0, 97.0]	3	11.9 [3.0, 37.0]	*0.075*
	18.5 - 24.9	171	84.1 [76.5, 89.6]	29	15.9 [10.4, 23.5]	
	25.0 - 29.9	140	79.4 [70.1, 86.4]	35	20.6 [13.6, 29.9]	
	30.0 - 39.9	157	90.7 [86.6, 93.7]	16	9.3 [6.3, 13.4]	
	40.0 - 80.0	36	92.5 [82.6, 97.0]	3	7.5 [3.0, 17.4]	
Education	None	6	66.5 [31.0, 90.0]	2	33.5 [10.3, 69.0]	*0.333*
	Primary (1-7 years)	41	94.5 [81.2, 98.5]	3	5.5 [1.5, 18.8]	
	Secondary (8-12 years)	297	84.0 [78.5, 88.4]	50	16.0 [11.6, 21.5]	
	Tertiary- certificate	131	85.8 [78.6, 90.9]	24	14.2 [9.1, 21.4]	
	Tertiary- Diploma/higher	51	86.7 [70.5, 94.7]	7	13.3 [5.3, 29.5]	
Married/Cohabiting	No	319	87.7 [83.8, 90.9]	43	12.3 [9.1, 16.3]	*0.122*
	Yes	207	82.1 [74.7, 87.7]	43	17.9 [12.3, 25.3]	
Income source[a]	Employed	145	88.9 [80.6, 94.0]	18	11.1 [6.0, 19.4]	***0.018***
	Spouse/partner	153	80.9 [72.6, 87.2]	34	19.1 [12.8, 27.4]	
	Parent/relative	72	75.9 [62.8, 85.4]	18	24.1 [14.6, 37.2]	
	Grant	150	91.3 [85.5, 94.9]	16	8.7 [5.1, 14.5]	
Household monthly	>R3200	215	82.0 [75.9, 86.8]	47	18.0 [13.2, 24.1]	*0.097*
	R3200 or less/none	310	87.5 [82.8, 91.1]	39	12.5 [9.0, 17.2]	

For peer review only - http://bmjopen.bmj.com/site/about/guidelines.xhtml

gross income						
Partner's HIV status	Negative	98	91.6 [85.8, 95.2]	12	8.4 [4.8, 14.2]	*0.081*
	Positive	243	85.0 [79.1, 89.4]	36	15.0 [10.6, 20.9]	
	Don't know	184	82.4 [76.4, 87.2]	38	17.6 [12.8, 23.6]	
Condom use frequency[a]	Never	43	85.9 [75.4, 92.3]	9	14.1 [7.7, 24.6]	*0.235*
	Sometimes	200	88.6 [82.6, 92.8]	26	11.4 [7.2, 17.4]	
	Always	280	83.2 [77.5, 87.6]	50	16.9 [12.4, 22.5]	
Planned pregnancy	No	289	86.2 [81.0, 90.2]	41	13.8 [9.8, 19.0]	*0.611*
	Yes	237	84.3 [77.9, 89.1]	45	15.7 [10.9, 22.1]	
Gestational age at ANC-1 visit	≤12 weeks	335	86.6 [81.8, 90.3]	44	13.4 [9.7, 18.2]	*0.443*
	13-20 weeks	136	83.2 [76.2, 88.5]	29	16.8 [11.6, 23.8]	
	>20 weeks	55	81.9 [70.9, 89.4]	13	18.1 [10.6, 29.1]	
Number of ANC visits[a]	0-4 visits	180	85.1 [79.5, 89.3]	30	14.9 [10.7, 20.5]	*0.921*
	5-12 visits	345	85.4 [81.1, 88.8]	56	14.6 [11.2, 18.9]	
**ART-related factors**						
Timing of HIV-positive result	Before pregnancy	394	87.3 [82.6, 90.9]	54	12.7 [9.1, 17.4]	*0.086*
	At ANC-1 or after	132	79.8 [71.1, 86.4]	32	20.2 [13.6, 28.9]	
Time since ART initiation	>12 months	392	87.5 [83.0, 90.9]	50	12.5 [9.1, 17.0]	***0.029***
	≤12 months	134	78.6 [70.3, 85.0]	36	21.4 [15.0, 29.7]	
Current ART regimen[a]	2nd/3rd line or unknown	107	92.3 [85.9, 95.9]	12	7.7 [4.1, 14.2]	***0.016***
	First-line	417	83.5 [79.5, 86.9]	74	16.5 [13.1, 20.5]	
Missed an ART dose last 7 days	No	501	85.7 [82.1, 88.6]	81	14.4 [11.4, 17.9]	*0.294*
	Yes	25	78.1 [56.0, 90.9]	5	21.9 [9.1, 44.1]	
Facing any ART adherence challenges	No	331	86.3 [81.3, 90.0]	45	13.8 [10.0, 18.7]	*0.551*
	Yes	195	83.7 [75.8, 89.4]	41	16.3 [10.6, 24.2]	
**Infant related factors (postpartum sample only)**						
	All	379	85.8 [81.4, 89.4]	57	14.2 [10.6, 18.6]	
Infant HIV status at enrolment	Negative	296	85.4 [80.3, 89.4]	49	14.6 [10.6, 19.7]	*0.882*
	Positive	9	88.3 [50.1, 98.3]	1	11.7 [1.7, 49.9]	
	Unknown	74	87.5 [72.6, 94.9]	7	12.5 [5.1, 27.5]	
Infant currently on ARV prophylaxis[a]	No	258	87.0 [81.8, 90.0]	35	13.0 [0.1, 18.2]	*0.326*
	Yes	119	83.5 [75.3, 89.4]	21	16.5 [10.7, 24.7]	
Infant ever breastfed[a]	No	124	82.3 [72.7, 89.1]	22	17.8 [11.0, 27.3]	*0.231*
	Yes	254	87.4 [82.5, 91.0]	35	12.6 [9.0, 17.5]	
Infant currently breastfeedin g	Yes	143	87.5 [81.4, 91.8]	20	12.5 [8.2, 18.6]	*0.427*
	No	111	87.2 [79.9, 92.1]	15	12.8 [7.9, 20.1]	
	Chose not to disclose	125	82.5 [73.0, 89.2]	22	17.5 [10.8, 27.0]	

For peer review only - http://bmjopen.bmj.com/site/about/guidelines.xhtml

Gestational age at birth[a]	≤37 weeks	56	83.1 [70.9, 90.8]	11	16.9 [9.2, 29.1]	*0.466*
	38 - 42 weeks	322	86.9 [81.9, 90.7]	45	13.1 [9.3, 18.1]	
Infant birth weight[a]	Birth weight≥2.5kg	345	86.6 [82.3, 89.9]	47	13.4 [10.1, 17.7]	*0.151*
	Low birth weight	33	78.4 [61.9, 89.1]	10	21.6 [10.9, 38.1]	

[a]denominator less than N due to missing responses; ANC-1 – antenatal care first visit; ARV – antiretroviral; BMI – body mass index; ART antiretroviral therapy; CI – confidence interval, VL – viral load. P-values are from a chi-squared test. Significant p-values <0.05 are in boldface font.

Factors associated with maternal viral load non-suppression

In univariable analyses to identify factors associated with mVL non-suppression amongst all women, independent variables with an overall odds ratio (OR) p-value<0.2 and included in the multivariable logistic regression model were: timing of first HIV-positive result, ART duration, ART regimen maternal age, BMI, marital status, household income level, partner's HIV status and condom use (**Table 3**). The OR p-values of other variables including that for the study group stratification were >0.2 and hence excluded from the adjusted model. All infant-related postnatal variables had OR p-values >0.2 and an adjusted model analyses was not conducted (**Table 4**).

In the adjusted model (**Table 3**), the odds of viral non-suppression were significantly increased amongst women who were on first-line ART regimen (adjusted odds ratio (AOR) 2.3 [95% CI: 1.1-4.6], p=0.025) and were married or cohabiting (AOR 2.0 [95% CI: 1.1-3.6], p=0.029). Weak associations were observed for increased odds of viral non-suppression amongst women who initiated ART within the most recent 12 months (AOR 1.9 [95% CI: 0.9-4.0], p=0.084) or did not know their male partner's HIV status (AOR 2.1 [95% CI: 1.0-4.7], p=0.065).

Compared to women aged 15-24 years, those aged 25 years or more had significantly reduced odds of viral non-suppression (AOR 0.4 [95% CI: 0.2-0.9], p=0.036 for 25-34 years and p=0.024 for 35-46 years). Extremely obese BMI of ≥40 was significantly associated with reduced odds of viral non-suppression compared to normal BMI of 18.5-24.9 (AOR 0.3 [95% CI: 0.1-0.9], p=0.029). There was no difference between normal BMI and either underweight, overweight or obese BMI.

Table 3: Factors associated with maternal viral load non-suppression among all women

	OR [95% CI]	p-value	AOR [95% CI]	p-value
Timing of HIV-positive result				
Before pregnancy	ref			

For peer review only - http://bmjopen.bmj.com/site/about/guidelines.xhtml

For peer review only

At ANC-1 or after	1.7 [0.9, 3.3]	0.088	0.9 [0.4,2.0]	0.730
Time since ART initiation				
>12 months	ref			
≤12 months	1.9 [1.1, 3.4]	**0.031**	1.9 [0.9,4.0]	0.084
Current ART regimen				
2nd/3rd line or unknown	ref			
First-line	2.4 [1.2, 4.8]	**0.018**	2.3 [1.1,4.6]	**0.025**
Age in years				
15 – 24	ref			
25 - 34	0.5 [0.3,1.1]	0.073	0.4 [0.2,0.9]	**0.036**
35 - 46	0.4 [0.2,0.9]	**0.029**	0.4 [0.2,0.9]	**0.024**
BMI				
18.5 - 24.9	ref			
13.0 - 18.4	0.7 [0.1, 3.8]	0.689	0.8 [0.2,4.4]	0.806
25.0 - 29.9	1.4 [0.7,2.8]	0.379	1.3 [0.6,2.9]	0.435
30.0 - 39.9	0.5 [0.3,1.0]	0.051	0.5 [0.3,1.1]	0.089
40.0 - 80.0	0.4 [0.2,1.2]	0.112	0.3 [0.1,0.9]	**0.029**
Married/Cohabiting				
No	ref			
Yes	1.6 [0.8, 2.8]	0.124	2.0 [1.1,3.6]	**0.029**
Partner's HIV status				
Negative	ref			
Positive	1.9 [0.9,4.3]	0.101	1.9 [0.9,4.1]	0.096
Don't know	2.3 [1.2,4.7]	**0.020**	2.1 [1.0,4.7]	0.065
Condom use frequency				
Never	ref			
Sometimes	0.8 [0.3,1.9]	0.579	0.8 [0.3,2.1]	0.627
Always	1.2 [0.6,2.6]	0.583	1.1 [0.6,2.1]	0.792
Household gross income/month				
>R3200	ref			
R3200 or less/none	0.6 [0.3, 1.1]	0.099	0.6 [0.4,1.1]	0.076

ANC-1 – antenatal care first visit; BMI – body mass index; ART- antiretroviral therapy; OR – Odds ratio; AOR – adjusted odds ratio; CI – confidence interval. Significant p-values <0.05 are in boldface font.

Table 4: Association between infant related postnatal factors and maternal viral load non-suppression

	OR [95% CI]	p-value
All		
Gestational age at birth		
≤37 weeks	ref	
38 - 42 weeks	0.7 [0.3, 1.7]	0.467

For peer review only

For peer review only - http://bmjopen.bmj.com/site/about/guidelines.xhtml

Infant birth weight		
Birth weight≥2.5kg	ref	
Low birth weight	1.8 [0.8, 4.0]	0.156
Infant HIV status at enrolment		
Negative	ref	
Positive	0.8 [0.1, 5.8]	0.797
Unknown	0.8 [0.3, 2.4]	0.736
Infant currently on ARV prophylaxis		
No	ref	
Yes	1.3 [0.4, 2.4]	0.327
Infant ever breastfed		
No	ref	
Yes	0.7 [0.3, 1.3]	0.233
Infant currently breastfeeding		
Yes	ref	
No	1.0 [0.5, 2.0]	0.935
Chose not to disclose	1.4 [0.7, 3.0]	0.265

ARV - antiretroviral; OR – Odds ratio; CI – confidence interval.

Discussion

The prevalence of mVL<1000 copies/mL in this rural South African district was estimated at 85.3%, and is still below the UNAIDS 2020 and 2030 targets of 90% and 95%, respectively, amongst persons on ART[15 16]. The observed VL suppression prevalence is higher than the 2017 South Africa national antenatal survey estimate of 79.5%, indicating that the district is comparatively performing well[24]. The postpartum study inclusion criteria of biological mothers could be biasing results towards women who frequently attend healthcare services with their biological children, and possibly under-estimating mVL non-suppression prevalence without those women who assign child postnatal care activities to other caregivers. The observed prevalence is comparable to PMTCT population in other African countries such as Malawi (84-88%), but is lower than that reported in an urban setting (91%) within South Africa, further supporting the existing inter-district disparities in PMTCT performance in-country[6 19 25]. These sub-national variations highlight the need for district-level surveys as opposed to provincial-level surveys which mask existing heterogeneity at district-level.

For peer review only - http://bmjopen.bmj.com/site/about/guidelines.xhtml

Even though the cross-sectional study design limited our understanding of causality, the significantly increased odds of having unsuppressed VL among women on first-line ART regimens could be related to the observed high proportion of unsuppressed VL amongst women who were on ART for ≤12 months. The extremely high self-reported ART well-adherent (95.2%) group likely includes a desirability bias, but other studies in African settings have shown reliability of self-reported ART adherence including its association with mVL[25 26]. Therefore, we conclude that the disproportionately high prevalence of unsuppressed mVL in the group on first-line ART regimen implies a combination of challenges with adherence amongst those recently initiated on ART and first-line regimen failure. Findings from Zimbabwe showed that being on second-line ART regimen versus first-line was associated with higher odds of viral suppression[27]. It is possible that regimen intervention or shift to second-line ART was delayed amongst some women. This is further supported by that three quarters of the study sample had been on ART for more than a year, and 90% had their first ANC visit early before 20 weeks gestation at which point over 70% already knew their HIV-positive status and were on ART.

One likely underlying driver of high mVL association with first-line ART is the 6-months long window recommended for following up those with low-level viraemia (50-1000 copies/mL), who however, are at risk of higher VL during this period. This study was conducted at the end of the national PMTCT guidelines practiced between 2015 and 2019 that recommended 6-monthly VL testing if a woman is virally suppressed (VL≤1000 copies/mL) or 4-6 weekly if their viral load is >1000 copies/mL[22]. The 6-months window was too long considering that it was used for low-level viraemia which may account for over 40% of early MTCT in some settings[3 6 25]. Given the new policies emphasizing targeting complete viral suppression of <50 copies/mL as opposed to VL<1000copies/mL, our results of 71.5% complete viral suppression shows that more work remains to be done[28]. The newly revised 2019 South African PMTCT guidelines have shortened the 6-months window to 8-10 weeks when VL is 50-999 copies/mL to address the problem of delayed VL and ART management, and have provided tips for the healthcare worker to understand barriers to ART adherence such as non-disclosure along with patient care interventions[28]. In addition, those newly initiated on ART are reviewed at 3 months regardless of baseline VL level, after which they are followed according to the universal schedule. These introduced shorter follow-up windows are expected to improve VL management going forward, particularly for those with recent ART initiation.

Periodic monitoring, evaluation and strengthening of these new guidelines as well as any outstanding service-delivery or user-related barriers is imperative. Client-related barriers and long turnaround times

For peer review only - http://bmjopen.bmj.com/site/about/guidelines.xhtml

for availability of VL results are examples of long-standing challenges that interfere with retention in care and timely follow-up management of VL and ART in LMIC settings[7 29-32]. In a 2018 evaluation of this district and five others, for example, we found that on average at district-level, between 37-100% of clinics delay (>7 days) returning VL results to PMTCT clients after the clinic has received them from the laboratory[33]. Given that the most cited potential barrier to ART adherence in this current study was time inconvenience, we hypothesise that this factor contributed to women delaying clinic attendance for their follow-up visits. At present the country is implementing an electronic monitoring system called E-labs to shorten the turnaround times along the VL processing pipeline from blood draw at clinics until availability of results at the testing clinic[34]. Mobile phone messaging directly to patients, such as MomConnect, should be incorporated into E-labs to shorten time to clinic-patient contact[35]. Point of care (POC) VL testing which is not common in this district should also be considered, given strong evidence for improved turnaround times when using POCs[36-38]. Alternatively, long-acting injectable antiretroviral therapeutics could ultimately reduce the burden of frequent clinic visits and daily pill adherence challenges, and thus studies developing these need to be strengthened earnestly[11].

The observed association of mVL non-suppression with young women aged 15-24 years is not unexpected. Earlier (2010-2014) studies, including a national PMTCT survey secondary analysis showed that adolescent PMTCT clients in South Africa are at higher risk of undesirable clinical and healthcare uptake outcomes[39 40]. Findings remain similar in the more recent (2016 - 2017) national representative data in South Africa where younger age was significantly associated with failure to achieve undetectable VL[24 41]. The revised PMTCT guidelines have included a guidance for adolescent-aware recommendations during antenatal and postpartum care. The guidance advises healthcare workers to identify psychosocial stressors and provide referrals for professional interventions, and emphasizes intensive adherence support such as enrolment into peer-led support groups[28]. Based on a systematic review of impactful interventions for improving healthcare uptake and adherence, family-centered initiatives could also be investigated for improving HIV health amongst pregnant and breastfeeding adolescents in this setting[42].

The high prevalence of unsuppressed VL observed amongst women who depended on another person including a spouse/partner for income aligns with the significantly increased odds amongst those who were married or cohabiting. The particularly high proportion (35%) of women who do not know their male partner's HIV status is also concerning and could be another underlying contributing factor. HIV disclosure to sexual partners, partner relations and partner involvement as determinants of utilization of HIV services

For peer review only - http://bmjopen.bmj.com/site/about/guidelines.xhtml

and HIV health outcomes amongst pregnant and postnatal women have been long-time challenges in African settings[43-46]. Non-disclosure of HIV status was the second highest cited potential barrier to ART adherence (11.7%) in this study. Data from pregnant women from the same province as this study district, collected seven years ago, indicated non-disclosure of HIV status as a key factor in poor ART adherence[47]. Disclosure to a partner has been associated with better HIV care outcomes in mothers and their babies in Rwanda[48]. An earlier study in Zimbabwe observed reduced uptake of HIV care among eligible PMTCT clients living with a male partner[46]. Given challenges involving male partners and particularly for younger women, the adolescent-aware recommendations in the current PMTCT guidelines do emphasize on sexual reproductive health counselling during ANC and identification of socio-contextual barriers such as abusive relationships to offer counselling referrals and ensure a safe and healthy home environment[28]. More work is however needed in this area.

The significant association between extreme BMI and reduced odds of VL non-suppression is not well understood and needs further investigation. A previous HIV vaccine efficacy trial reported an association between overweight/obese BMI and suppressed vaccine-induced immune response against HIV in a South African population[49]. Other separate studies on HIV-positive persons from different countries, initiated on antiretroviral treatment, found high BMI to be associated with better CD4 count recovery[50-52]. Whether our findings are simply due to the increased prevalence of obesity in South Africa (with women ranking in the highest burdened countries), and/or increased weight gain due to antiretrovirals as previously implied or there is an underlying biological mechanism, is unclear and needs further investigation[53-55].

Limitations

Although the 3-month recruitment period was expected to achieve the desired sample size, assuming 50% participation rate, the realized sample sizes were not as high as planned. However, there was no statistically significant heterogeneity in the primary outcome between the study groups, making the survey-weighted results reliable. The reduced sample size in the postpartum groups could have resulted from the study's inclusion criterion of biological mother-infant pairs. It is common practice locally to assign child healthcare visits to other caregivers. The 15-26 weeks postpartum study group had the lowest sample size which we attribute to lack of routine childhood vaccination points during this child growth period. Although the findings of our sample could have introduced a selection bias, the viral load

For peer review only - http://bmjopen.bmj.com/site/about/guidelines.xhtml

suppression estimates lie within the range of other estimates reported in the country. Desirability bias from self-reported adherence to ART and condom use and knowing of male partner's HIV status, cannot be ruled out completely. Causality could not be inferred in this cross-sectional study design and a prospective cohort approach would be needed to confirm some of the associations.

The sample was designed to measure prevalence of VL>1000 copies/mL and the data were not sufficient to investigate differences between VL stages within the 50-1000 copies/mL range to fully inform the newly implemented guidelines. Virological failure and treatment failure could not be confirmed with certainty given only one VL measurement done.

Conclusions

The UNAIDS 2020/2030 targets for viral suppression amongst PMTCT clients in this district have not been achieved. This study highlights that socio-demographic factors (maternal age, BMI and marital/cohabiting status) and type of ART regimen disproportionately influence maternal VL suppression. While the role of BMI requires further investigation, recommendations outlined in the current South African PMTCT guidelines have potential to mitigate other identified factors through the improved schedules for monitoring VL and ART and targeted psycho-socio support for younger women with consideration of marital/male partner influence. Given that low-level viraemia may also increase the risk of vertical HIV transmission, it is imperative to periodically monitor, evaluate and strengthen the implementation of these guidelines alongside complementary district-level interventions, to keep all women virally suppressed.

For peer review only

Declarations

Acknowledgements: Authors would like to thank: the Ehlanzeni district PMTCT leadership and partners for supporting the placement of the study at the clinics; the Ehlanzeni local Rob Ferreira laboratory for handling of blood samples; the staff at NICD for conducting laboratory assays for viral load measurement; the SAMRC data collection and coordinating team; and Dr Witness Chirinda for involvement in study planning discussions and contributing to data cleaning.

For peer review only - http://bmjopen.bmj.com/site/about/guidelines.xhtml

Availability of data and materials: The dataset supporting the conclusions of this article will be available after the primary objectives for the study have been published. Interested researchers should contact the corresponding author to confirm if data have become open access.

Competing interests: The authors declare that they have no financial or personal relationship(s) that may have inappropriately influences them in writing this article.

Funding & disclaimer: This study was funded by the National Department of Health – South Africa. Authors NKN & AEG were supported by the South African Medical Research Council. Author TEM was supported by the National Research Foundation – South Africa. AJP is funded by Wellcome (grant 108065/Z/15/Z). The views expressed in this manuscript are our own and not an official position of the institution or funder.

Authors' contributions: Study conceptualization and design **–** AEG, CW, APr, CJL, NKN, PvDP, TT. Data analyses – NKN, CJL. First draft of manuscript- NKN, TEM. Laboratory assays provider: APu. Critical review and revision of subsequent drafts and approval of vinal version: all authors.

References

1. UNICEF, UNAIDS. Women: At the heart of the response for children. https://data.unicef.org/resources/women-at-the-heart-of-the-hiv-response-for-children/, 2018.
2. Goga A, Chirinda W, Ngandu N, et al. Closing the gaps to eliminate mother-to-child transmission of HIV (MTCT) in South Africa: Understanding MTCT case rates, factors that hinder the monitoring and attainment of targets, and potential game changers. SAMJ 2018;**108**(3 Suppl 1):S17-S24
3. Goga AE, Van de Perre P, Ngandu N, et al. Eliminating HIV transmission through breast milk from women taking antiretroviral drugs. Bmj 2021;**374**:n1697 doi: 10.1136/bmj.n1697
4. Goga A, Lombard C, Jackson D, et al. Impact of breastfeeding, maternal antiretroviral treatment and health service factors on 18-month vertical transmission of HIV and HIV-free survival: Results from a nationally representative HIV-exposed infant cohort, South Africa. Journal of Epidemiology and Community Health 2020;**0**:1-9
5. Technau K-G, Kalk E, Coovadia A, et al. Timing of maternal HIV testing and uptake of Prevention of Mother-to-Child Transmission interventions among women and their infected infants in Johannesburg, South Africa. Journal of Acquired Immune Deficiency Syndrome 2014;**65**(5):e170-e78
6. Myer L, Phillips TK, McIntyre JA, et al. HIV viraemia and mother-to-child transmission risk after antiretroviral therapy initiation in pregnancy in Cape Town, South Africa. HIV Med 2017;**18**(2):80-88 doi: 10.1111/hiv.12397
7. Glass TR, Motaboli L, Nsakala B, et al. The viral load monitoring cascade in a resource-limited setting: A prospective multicentre cohort study after introduction of routine viral load monitoring in rural Lesotho. PLoS One 2019;**14**(8):e0220337 doi: 10.1371/journal.pone.0220337

For peer review only - http://bmjopen.bmj.com/site/about/guidelines.xhtml

8. Ngarina M, Kilewo C, Karlsson K, et al. Virologic and immunologic failure, drug resistance and mortality during the first 24 months postpartum among HIV-infected women initiated on antiretroviral therapy for life in the Mitra plus Study, Dar es Salaam, Tanzania. BMC Infect Dis 2015;**15**:175 doi: 10.1186/s12879-015-0914-z
9. Nyakura J, Shewade HD, Ade S, et al. Viral load testing among women on 'option B+' in Mazowe, Zimbabwe: How well are we doing? PLoS One 2019;**14**(12):e0225476 doi: 10.1371/journal.pone.0225476
10. Erlwanger AS, Joseph J, Gotora T, et al. Patterns of HIV Care Clinic Attendance and Adherence to Antiretroviral Therapy Among Pregnant and Breastfeeding Women Living With HIV in the Context of Option B+ in Zimbabwe. J Acquir Immune Defic Syndr 2017;**75 Suppl 2**:S198-S206 doi: 10.1097/QAI.0000000000001347
11. Van de Perre P, Goga A, Ngandu N, et al. Eliminating postnatal HIV transmission in high incidence areas: need for complementary biomedical interventions. Lancet 2021;**397**(10281):1316-24 doi: 10.1016/S0140-6736(21)00570-5
12. Waitt C, Low N, Van de Perre P, et al. Does U=U for breastfeeding mothers and infants? Breastfeeding by mothers on effective treatment for HIV infection in high-income settings. The lancet HIV 2018;**5**:e531–36
13. Woldesenbet SA, Kufa T, Lombard C, et al. The 2017 National Antenatal Sentinel HIV Survey, South Africa: National Department of Health South Africa, National Institute of Communicable Diseases, 2019.
14. World Health Organization. Global guidance on processes and criteria for validation: Elimination of mother-to-child transmission of HIV and syphilis: second edition. 2017. https://apps.who.int/iris/bitstream/handle/10665/259517/9789241513272-eng.pdf Geneva, 2017.
15. UNAIDS. 90-90-90. An ambitious treatment target to help end the AIDS epidemic. http://files.unaids.org/en/media/unaids/contentassets/documents/unaidspublication/2014/90-90-90_en.pdf: United Nations AIDS, 2014.
16. UNAIDS. FAST-TRACK. Ending the AIDS epidemic by 2030. https://www.unaids.org/sites/default/files/media_asset/JC2686_WAD2014report_en.pdf: United Nations AIDS, 2014.
17. Massyn N, Peer N, Padarath A, et al. District Health Barometer 2015/16. http://www.hst.org.za Health Systems Trust, 2016.
18. Clouse K, Schwartz S, Van Rie A, et al. "What they wanted was to give birth; nothing else": barriers to retention in option B+ HIV care among postpartum women in South Africa. J Acquir Immune Defic Syndr 2014;**67**(1):e12-8 doi: 10.1097/QAI.0000000000000263
19. Hosseinipour M, Nelson JAE, Trapence C, et al. Viral Suppression and HIV Drug Resistance at 6 Months Among Women in Malawi's Option B+ Program: Results From the PURE Malawi Study. J Acquir Immune Defic Syndr 2017;**75 Suppl 2**:S149-S55 doi: 10.1097/QAI.0000000000001368
20. Kendall C, Claessens L, Dorward J, et al. Reasons for failure of prevention of mother-to-child HIV transmission in a rural South African district hospital Southern African Journal of HIV Medicine 2015;**16**(1)
21. Myer L, Dunning L, Lesosky M, et al. Frequency of Viremic Episodes in HIV-Infected Women Initiating Antiretroviral Therapy During Pregnancy: A Cohort Study. Clinical infectious diseases : an official publication of the Infectious Diseases Society of America 2017;**64**(4):422-27 doi: 10.1093/cid/ciw792
22. South Africa National Department of Health. National Consolidated Guidelines for the Prevention of Mother-to-Child Transmission of HIV (PMTCT) and the management of HIV in children, adolescents and adults. https://www.childrenandaids.org/sites/default/files/2017-

For peer review only - http://bmjopen.bmj.com/site/about/guidelines.xhtml

04/SouthAfrica_National-Consolidated-HIV_2015.pdf National Department of Health, South Africa, 2015.
23. Harris PA, Taylor R, Thielke R, et al. Research electronic data capture (REDCap)--a metadata-driven methodology and workflow process for providing translational research informatics support. Journal of biomedical informatics 2009;**42**(2):377-81 doi: 10.1016/j.jbi.2008.08.010
24. Woldesenbet SA, Kufa T, Barron P, et al. Viral suppression and factors associated with failure to achieve viral suppression among pregnant women in South Africa. AIDS (London, England) 2020;**34**(4):589-97 doi: 10.1097/QAD.0000000000002457
25. Landes M, van Lettow M, Nkhoma E, et al. Low detectable postpartum viral load is associated with HIV transmission in Malawi's prevention of mother-to-child transmission programme. J Int AIDS Soc 2019;**22**(6):e25290 doi: 10.1002/jia2.25290
26. Phillips TK, Wilson IB, Brittain K, et al. Decreases in Self-Reported ART Adherence Predict HIV Viremia Among Pregnant and Postpartum South African Women. J Acquir Immune Defic Syndr 2019;**80**(3):247-54 doi: 10.1097/QAI.0000000000001909
27. Bvochora T, Satyanarayana S, Takarinda KC, et al. Enhanced adherence counselling and viral load suppression in HIV seropositive patients with an initial high viral load in Harare, Zimbabwe: Operational issues. PLoS One 2019;**14**(2):e0211326 doi: 10.1371/journal.pone.0211326
28. South African National Department of Health. Guideline for the Prevention of Mother to Child Transmission of Communicable Infections. https://www.knowledgehub.org.za/elibrary/guideline-prevention-mother-child-transmission-communicable-infections National Department of Health, South Africa, October 2019.
29. Shiferaw MB, Yismaw G. Magnitude of delayed turnaround time of laboratory results in Amhara Public Health Institute, Bahir Dar, Ethiopia. Clinical microbiology reviews 2019;**19**(1):240 doi: 10.1128/cmr.00097-18

10.1186/s12913-019-4077-2
30. Fox MP, Brennan AT, Nattey C, et al. Delays in repeat HIV viral load testing for those with elevated viral loads: a national perspective from South Africa. J Int AIDS Soc 2020;**23**(7):e25542 doi: 10.1002/jia2.25542
31. Kroidl A, Burger T, Urio A, et al. High turnaround times and low viral resuppression rates after reinforced adherence counselling following a confirmed virological failure diagnostic algorithm in HIV-infected patients on first-line antiretroviral therapy from Tanzania. Tropical medicine & international health : TM & IH 2020 doi: 10.1111/tmi.13373
32. Tenthani L, Haas AD, Tweya H, et al. Retention in care under universal antiretroviral therapy for HIV-infected pregnant and breastfeeding women ('Option B+') in Malawi. AIDS (London, England) 2014;**28**(4):589-98 doi: 10.1097/QAD.0000000000000143
33. Ngandu NK, Nsibande DF, Magasana V, et al. Quality and turnaround times of PMTCT viral load monitoring under Option B+ in six South African districts with high antenatal HIV burden. South African Medical Journal 2021;**111**(8):759-67 doi: https://doi.org/10.7196/SAMJ.2021.v111i8.15496
34. Chugudu K, Kirsten T, Gorogodo B, et al. Demonstration of usability of a digital intervention (DHI) for strengthening the clinic: lab interface for HIV Viral Load testing in a South African clinical setting (Elabs). 9th South African AIDS Conference. Durban South Africa, 2019.
35. Peter J, Barron P, Pillay Y. Using mobile technology to improve maternal, child and youth health and treatment of HIV patients. South African Medical Journal 2016;**106**(1) doi: 10.7196/SAMJ.2016.v106i1.10209

For peer review only - http://bmjopen.bmj.com/site/about/guidelines.xhtml

36. Lesosky M, Glass T, Mukonda E, et al. Optimal timing of viral load monitoring during pregnancy to predict viraemia at delivery in HIV-infected women initiating ART in South Africa: a simulation study. J Int AIDS Soc 2017;**20 Suppl 7** doi: 10.1002/jia2.25000
37. Poulet E, Wolters L, Wapling J, et al. Point-of-Care HIV Viral Load Testing: an Essential Tool for a Sustainable Global HIV/AIDS Response. J Int AIDS Soc 2019;**32**(3) doi: 10.1002/jia2.2538710.1128/cmr.00097-18
38. World Health Organization. Updated recommendations on HIV prevention, Infant diagnosis, Antiretroviral initiation and Monitoring. https://apps.who.int/iris/bitstream/handle/10665/340190/9789240022232-eng.pdf?sequence=1&isAllowed=y, 2021.
39. Ramraj T, Jackson D, Dinh TH, et al. Adolescent Access to Care and Risk of Early Mother-to-Child HIV Transmission. The Journal of adolescent health : official publication of the Society for Adolescent Medicine 2018;**62**(4):434-43 doi: 10.1016/j.jadohealth.2017.10.007
40. Fatti G, Shaikh N, Eley B, et al. Adolescent and young pregnant women at increased risk of mother-to-child transmission of HIV and poorer maternal and infant health outcomes: A cohort study at public facilities in the Nelson Mandela Bay Metropolitan district, Eastern Cape, South Africa. South African medical journal = Suid-Afrikaanse tydskrif vir geneeskunde 2014;**104**(12):874-80 doi: 10.7196/samj.8207
41. Moyo F, Mazanderani AH, Murray T, et al. Achieving maternal viral load suppression for elimination of mother-to-child transmission of HIV in South Africa. AIDS (London, England) 2021;**35**(2):307-16 doi: 10.1097/QAD.0000000000002733
42. Vrazo AC, Firth J, Amzel A, et al. Interventions to significantly improve service uptake and retention of HIV-positive pregnant women and HIV-exposed infants along the prevention of mother-to-child transmission continuum of care: systematic review. Tropical medicine & international health : TM & IH 2018;**23**(2):136-48 doi: 10.1111/tmi.13014
43. Hampanda KM. Intimate partner violence and HIV-positive women's non-adherence to antiretroviral medication for the purpose of prevention of mother-to-child transmission in Lusaka, Zambia. Social science & medicine 2016;**153**:123-30 doi: 10.1016/j.socscimed.2016.02.011
44. Hodgson I, Plummer ML, Konopka SN, et al. A systematic review of individual and contextual factors affecting ART initiation, adherence, and retention for HIV-infected pregnant and postpartum women. PLoS One 2014;**9**(11):e111421 doi: 10.1371/journal.pone.0111421
45. Ashaba S, Kaida A, Coleman JN, et al. Psychosocial challenges facing women living with HIV during the perinatal period in rural Uganda. PLoS One 2017;**12**(5):e0176256 doi: 10.1371/journal.pone.0176256
46. Muchedzi A, Chandisarewa W, Keatinge J, et al. Factors associated with access to HIV care and treatment in a prevention of mother to child transmission programme in urban Zimbabwe. J Int AIDS Soc 2010;**13**:38 doi: 10.1186/1758-2652-13-38
47. Ramlagan S, Peltzer K, Ruiter RAC, et al. Prevalence and Factors Associated with Fixed-Dose Combination Antiretroviral Drugs Adherence among HIV-Positive Pregnant Women on Option B Treatment in Mpumalanga Province, South Africa. International journal of environmental research and public health 2018;**15**(1) doi: 10.3390/ijerph15010161
48. Maurice Bucagu JdDB, John Muganda, Claire Perrine Humblet. Socio-economic, clinical and biological risk factors for mother - to - child transmission of HIV-1 in Muhima health centre (Rwanda): a prospective cohort study. Archives of Public Health 2013;**71**(4)
49. Hopkins KL, Laher F, Otwombe K, et al. Predictors of HVTN 503 MRK-AD5 HIV-1 gag/pol/nef vaccine induced immune responses. PLoS One 2014;**9**(8):e103446 doi: 10.1371/journal.pone.0103446

For peer review only - http://bmjopen.bmj.com/site/about/guidelines.xhtml

50. Li X, Ding H, Geng W, et al. Predictive effects of body mass index on immune reconstitution among HIV-infected HAART users in China. BMC Infect Dis 2019;**19**(1):373 doi: 10.1186/s12879-019-3991-6
51. Blashill AJ, Mayer KH, Crane HM, et al. Body mass index, immune status, and virological control in HIV-infected men who have sex with men. Journal of the International Association of Providers of AIDS Care 2013;**12**(5):319-24 doi: 10.1177/2325957413488182
52. Koethe JR, Jenkins CA, Lau B, et al. Body mass index and early CD4 T-cell recovery among adults initiating antiretroviral therapy in North America, 1998-2010. HIV Med 2015;**16**(9):572-7 doi: 10.1111/hiv.12259
53. Bares SH, Smeaton LM, Xu A, et al. HIV-Infected Women Gain More Weight than HIV-Infected Men Following the Initiation of Antiretroviral Therapy. Journal of women's health 2018;**27**(9):1162-69 doi: 10.1089/jwh.2017.6717
54. Cois A, Day C. Obesity trends and risk factors in the South African adult population. BMC obesity 2015;**2**:42 doi: 10.1186/s40608-015-0072-2
55. The GBD 2015 Obesity Collaborators. Health Effects of overweight and Obesity in 195 ountries over 25 Years. The New England Journal of Medicine 2017;**377**(1)

Supplementary files

Supplementary File 1

Title: STROBE 2007 (v4) Statement—Checklist of items that should be included in reports of *cross-sectional studies*
Format: pdf, table

Supplementary File 2

Title: Summary of participant characteristics by maternal viral load non-suppression per study group.

Format: pdf, table

For peer review only

STROBE 2007 (v4) Statement—Checklist of items that should be included in reports of *cross-sectional studies*

**Title and abstract**	1	(*a*) Indicate the study's design with a commonly used term in the title or the abstract	1
		(*b*) Provide in the abstract an informative and balanced summary of what was done and what was found	2
**Introduction**			
Background/rationale	2	Explain the scientific background and rationale for the investigation being reported	4
Objectives	3	State specific objectives, including any prespecified hypotheses	5
**Methods**			
Study design	4	Present key elements of study design early in the paper	5
Setting	5	Describe the setting, locations, and relevant dates, including periods of recruitment, exposure, follow-up, and data collection	5
Participants	6	(*a*) Give the eligibility criteria, and the sources and methods of selection of participants	5
Variables	7	Clearly define all outcomes, exposures, predictors, potential confounders, and effect modifiers. Give diagnostic criteria, if applicable	6, 7
Data sources/ measurement	8*	For each variable of interest, give sources of data and details of methods of assessment (measurement). Describe comparability of assessment methods if there is more than one group	6, 7
Bias	9	Describe any efforts to address potential sources of bias	7
Study size	10	Explain how the study size was arrived at	5
Quantitative variables	11	Explain how quantitative variables were handled in the analyses. If applicable, describe which groupings were chosen and why	6, 7
Statistical methods	12	(*a*) Describe all statistical methods, including those used to control for confounding	7
		(*b*) Describe any methods used to examine subgroups and interactions	
		(*c*) Explain how missing data were addressed	6,7
		(*d*) If applicable, describe analytical methods taking account of sampling strategy	7
		(*e*) Describe any sensitivity analyses	
**Results**			

For peer review only

Participants	13*	(a) Report numbers of individuals at each stage of study—eg numbers potentially eligible, examined for eligibility, confirmed eligible, included in the study, completing follow-up, and analysed	7
		(b) Give reasons for non-participation at each stage	
		(c) Consider use of a flow diagram	
Descriptive data	14*	(a) Give characteristics of study participants (eg demographic, clinical, social) and information on exposures and potential confounders	7-10
		(b) Indicate number of participants with missing data for each variable of interest	
Outcome data	15*	Report numbers of outcome events or summary measures	
Main results	16	(*a*) Give unadjusted estimates and, if applicable, confounder-adjusted estimates and their precision (eg, 95% confidence interval). Make clear which confounders were adjusted for and why they were included	11-16
		(*b*) Report category boundaries when continuous variables were categorized	
		(*c*) If relevant, consider translating estimates of relative risk into absolute risk for a meaningful time period	
Other analyses	17	Report other analyses done—eg analyses of subgroups and interactions, and sensitivity analyses	
**Discussion**			
Key results	18	Summarise key results with reference to study objectives	17
Limitations	19	Discuss limitations of the study, taking into account sources of potential bias or imprecision. Discuss both direction and magnitude of any potential bias	3, 20
Interpretation	20	Give a cautious overall interpretation of results considering objectives, limitations, multiplicity of analyses, results from similar studies, and other relevant evidence	20
Generalisability	21	Discuss the generalisability (external validity) of the study results	20
**Other information**			
Funding	22	Give the source of funding and the role of the funders for the present study and, if applicable, for the original study on which the present article is based	21

*Give information separately for cases and controls in case-control studies and, if applicable, for exposed and unexposed groups in cohort and cross-sectional studies.

Note: An Explanation and Elaboration article discusses each checklist item and gives methodological background and published examples of transparent reporting. The STROBE checklist is best used in conjunction with this article (freely available on the Web sites of PLoS Medicine at http://www.plosmedicine.org/, Annals of Internal Medicine at http://www.annals.org/, and Epidemiology at http://www.epidem.com/). Information on the STROBE Initiative is available at www.strobe-statement.org.

For peer review only

Supplementary file 1: Summary of participant characteristics by maternal viral load non-suppression per study group

	Third trimester		0-14 weeks postpartum		15-26 weeks postpartum		27-52 weeks postpartum		53-104 weeks postpartum	
	N	% VL>1000 [95% CI]	N	% VL>1000 [95% CI]	N	% VL>1000 [95% CI]	N	% VL>1000 [95% CI]	N	% VL>1000 [95% CI]
All	176	17.1 [11.7, 24.2]	128	15.0 [8.5, 25.2]	75	6.9 [2.2, 20.1]	123	17.5 [9.0, 31.2]	110	16.4 [9.3, 27.2]
**Socio-demographics and antenatal care**										
Age in years										
15 – 24	33	[illegible] [13.2, 51.6]	23	3.1 [0.2, 28.9]	12	16.9 [1.2, 76.6]	17	28.0 [10.4, 56.5]	20	39.0 [25.9, 54.0]
25 - 34	99	15.4 [7.7, 28.4]	75	17.0 [6.8, 36.4]	48	6.0 [1.5, 21.5]	73	16.7 [6.3, 37.4]	56	14.5 [4.4, 38.3]
35 - 46	44	12.1 [3.2, 36.8]	30	20.2 [7.3, 44.9]	15	0.0	33	14.2 [3.9, 40.8]	34	5.3 [1.2, 20.6]
P-values		*0.332*		*[illegible]*		*0.471*		*0.538*		***0.016***
BMI										
13.0 to 24.9	33	12.1 [3.2, 36.4]	48	19.1 [5.7, 47.8]	[illegible]	12.7 [4.2, 32.6]	53	12.8 [6.2, 24.4]	53	19.0 [8.1, 38.6]
25 to 29.9	55	25.9 [15.1, 40.7]	41	20.8 [6.4, 50.3]	19	[illegible]	39	25.2 [7.0, 60.3]	21	23.3 [12.2, 39.9]
30 to 76.7	86	13.9 [7.9, 23.2]	39	2.1 [0.2, 19.3]	22	0.0	30	16.7 [illegible]	35	9.0 [3.4, 21.4]
P-values		*0.175*		*0.274*		*0.115*		*0.351*		*0.184*
Education										
>12 years of education	59	17.9 [6.3, 41.6]	59	13.1 [6.7, 24.0]	23	3.9 [0.6, 21.3]	43	22.1 [9.4, 43.7]	29	8.6 [1.3, 40.3]
≤12 years of education	117	16.7 [9.7, 27.2]	69	16.4 [6.9, 34.1]	52	8.1 [2.4, 24.0]	80	14.9 [8.8, 23.9]	81	19.3 [10.6, 32.4]
P-values		*0.899*		*0.644*		*0.335*		*0.192*		*0.324*
Married/Cohabiting										
No	114	15.3 [8.0, 27.3]	77	8.2 [4.0, 16.2]	46	1.9 [0.2, 17.2]	68	18.5 [11.1, 29.2]	57	16.1 [7.8, 30.2]

For peer review only - http://bmjopen.bmj.com/site/about/guidelines.xhtml

Yes	62	20.7 [11.1, 35.2]	51	24.5 [9.5, 49.9]	29	13.1 [4.7, 31.6]	55	16.4 [3.8, 49.0]	53	16.6 [7.9, 31.6]
P-values		*0.480*		*0.083*		***0.045***		*0.846*		*0.929*
Income source[a]										
Employed	47	13.4 [6.1, 27.1]	41	8.6 [2.3, 27.4]	22	0.0	26	11.6 [2.7, 37.9]	27	22.0 [5.0, 60.3]
Spouse/partner	54	20.5 [9.3, 39.1]	37	24.3 [6.3, 60.5]	19	19.0 [6.5, 44.0]	40	18.8 [4.5, 53.5]	37	15.4 [6.9, 30.6]
Parent/relative	34	[illegible]4.2 [7.0, 5[illegible].4]	17	30.1 [9.5, 63.8]	9	10.3 [0.6, 69.5]	14	24.4 [8.1, 54.2]	16	26.7 [6.8, 64.5]
Grant	38	12.7 [3.9, 33.8]	32	4.7 [0.9, 20.2]	24	0.0	43	17.3 [7.0, 36.8]	29	9.0 [1.7, 35.9]
P-values		*0.580*		*0.135*		*0.146*		*0.754*		*0.513*
Household gross income/month										
>R3200	72	21.7 [15.4, 29.7]	60	15.1 [6.0, [illegible]3.1]	34	5.2 [1.4, 18.0]	55	27.2 [14.6, 45.0]	41	18.7 [6.3, 43.9]
R3200 or less/none	104	13.4 [6.8, 24.9]	68	14.9 [7.6, 27.2]	41	8.1 [1.5, 33.1]	68	9.8 [5.6, 16.4]	68	15.5 [8.3, 27.0]
P-values		*0.168*		*0.983*		*0.642*		***0.007***		*0.711*
Partner's HIV status[a]										
Negative	30	24.1 [13.1, 40.1]	17	3.7 [0.2, 37.5]	15	0.0	28	11.4 [4.4, 26.5]	20	5.1 [0.4, 41.2]
Positive	71	11.8 [3.3, 34.2]	53	18.1 [7.8, 36.8]	38	14.7 [5.1, 35.5]	6[illegible]	17.7 [9.[illegible], 31.0]	57	12.5 [3.9, 33.1]
Don't know	75	19.2 [10.3, 32.9]	57	16.4 [6.3, 36.5]	22	0.0	35	21.2 [7.7, 46.6]	33	27.3 [20.2, 35.7]
P-values		*0.429*		*0.354*		***0.035***		*0.429*		*0.137*
Condom use frequency[a]										
Never	26	23.5 [12.8, 39.2]	11	11.8 [2.0, 46.4]	1	0.0	7	0.0	7	18.2 [4.2, 53.1]
Sometimes	72	17.4 [9.0, 30.9]	46	14.1 [2.4, 52.3]	24	3.1 [0.3, 23.2]	39	15.7 [8.3, 27.9]	45	6.1 [2.3, 15.3]

For peer review only - http://bmjopen.bmj.com/site/about/guidelines.xhtml

Always	78	15.0 [7.0, 29.4]	70	16.5 [8.1, 30.7]	49	9.3 [2.6, 28.4	75	19.7 [7.7, 42.0]	58	22.3 [13.9, 33.8]
P-values		*0.560*		*0.920*		*0.582*		*0.501*		***0.006***
Planned pregnancy										
No	99	13.1 [6.2, 25.6]	75	14.3 [4.6, 36.6]	44	8.8 [2.2, 28.8]	58	19.9 [11.6, 31.9]	54	13.5 [5.7, 28.5]
Yes	77	22.0 [14.0, 32.9]	53	16.0 [6.9, 32.9]	31	4.3 [0.7, 21.0]	65	15.3 [4.1, 43.6]	56	18.6 [9.3, 33.5]
P-values		*0.210*		*0.871*		*0.444*		*0.646*		*0.479*
Gestation at ANC-1 visit										
≤12 weeks	75	12.7 [4.6, 30[illegible]]	86	13.1 [4.7, 31.6]	56	6.4 [1.5, 23.2]	84	19.1 [9.2, 35.5]	78	15.2 [8.0, 26.8]
13-20 weeks	66	21.1 [13.8, 30.9]	33	12.7 [8.[illegible], 38.8]	11	13.8 [2.2, 53.6]	29	8.3 [1.6, 32.6]	26	24.8 [12.9, 42.5]
>20 weeks	35	19.7 [8.6, 39.0]	9	34.3 [15.1, [illegible]0.4]	8	0.0	10	29.9 [5.2, 76.9]	6	0.0 [0.0, 0.0]
P-values		*0.442*		*0.276*		*0.418*		*0.362*		*0.201*
Number of ANC visits[a]										
0-4 visits	93	16.7 [8.3, 30.8]	34	20.9 [9.3, 40.5]	17	4.6 [0.5, 3[illegible].7]	33	14.9 [4.6, 38.6]	33	14.1 [6.9, 26.6]
5-12 visits	83	17.5 [12.6, 23.7]	93	12.8 [7.2, 21.6]	58	7.6 [2.1, 23.7]	90	18.5 [9.4, 33.2]	77	17.5 [8.8, 31.6]
P-values		*0.893*		*0.139*		*0.646*		*0.653*		*0.550*
**Primary exposure variables**										
Timing of HIV-positive result										
Before pregnancy	128	14.2 [7.4, 25.6]	81	11.1 [5.6, 20.8]	59	7.8 [2.6, 21.5]	95	15.6 [5.9, 35.2]	85	14.0 [6.3, 28.4]
At ANC-1 or after	48	24.8 [17.2, 34.3]	47	22.0 [7.0, 51.3]	16	4.3 [0.3, 43.4]	28	23.8 [8.3, 52.1]	25	23.7 [11.9, 41.5]
P-values		*0.108*		*0.296*		*0.607*		*0.523*		*0.231*
Time since ART initiation										
>12 months	97	9.8	75	9.6	61	7.5	102	15.9	107	15.9

For peer review only - http://bmjopen.bmj.com/site/about/guidelines.xhtml

For peer review only

		[5.5, 16.8]		[5.5, 16.2]		[2.4, 21.0]		[6.5, 33.9]		[8.7, 27.4]
≤12 months	79	25.8 [19.9, 32.7]	53	22.7 [9.7, 44.6]	14	4.9 [0.3, 47.6]	*21*	24.8 [6.7, 60.2]	3	34.2 [8.5, 74.3]
P-values		***0.001***		*0.093*		*0.715*		*0.539*		*0.196*
Current ART regimen[a]										
2nd/3rd line or unknown	28	21.2 [9.3, 41.5]	29	5.4 [1.1, 22.7]	14	4.8 [0.4, 36.1]	26	3.4 [0.2, 37.3]	22	8.6 [1.9, 31.2]
First line	147	16.5 [illegible]	99	17.6 [9.9, 29.2]	61	7.5 [1.9, 25.6]	97	21.6 [12.6, 34.3]	87	18.3 [10.1, 30.8]
P-values		*[illegible]*		*0.136*		*0.717*		***0.048***		*0.251*
Missed an ART dose last 7 days										
No	168	16.7 [11.3, 24.0]	[illegible]	12.2 [illegible]	72	7.2 [2.3, 20.5]	116	18.6 [9.8, 32.4]	103	16.3 [9.0, 27.5]
Yes	8	26.3 [5.3, 69.3]	5	[illegible]	3	0.0	7	0.0	7	18.2 [4.2, 53.1]
P-values		*0.455*		***[illegible]***		*0.545*		*0.066*		*0.850*
Facing any ART adherence challenges										
No	121	12.6 [7.2, 21.2]	60	17.3 [7.9, 33.7]	43	6.4 [illegible]	72	14.7 [8.1, 25.1]	80	16.7 [8.3, 30.6]
Yes	55	25.0 [13.9, 40.8]	68	12.2 [5.4, 25.1]	32	[illegible]	51	21.1 [5.3, 56.1]	30	15.5 [4.6, 41.1]
P-values		*0.078*		*0.503*		*0.863*		*0.616*		*0.903*
**Infant related factors (postpartum sample only)**										
All			*127*	*15.0 [8.5, 25.2]*	*75*	*6.9 [2.2, 20.1]*	*123*	*[illegible]*	*110*	*16.4 [9.3, 27.2]*
Infant ever breastfed										
No			49	25.1 [8.7, 54.1]	21	3.0 [0.4, 21.4]	42	22.2 [9.3, 44.1]	34	14.3 [5.4, 32.7]
Yes			79	8.3 [3.7, 17.6]	53	8.3 [2.4, 25.3]	81	15.3 [7.9, 27.6]	76	17.2 [9.0, 30.3]
P-values				*0.138*		*0.355*		*0.268*		*0.668*
Infant currently breastfeeding										

For peer review only - http://bmjopen.bmj.com/site/about/guidelines.xhtml

Yes		60	9.4 [3.7, 21.8]	35	10.2 [3.2, 28.1]	48	14.4 [5.7, 32.0]	20	19.6 [9.3, 36.7]
No		19	5.3 [0.7, 32.1]	18	4.3 [0.3, 39.7]	33	16.5 [9.4, 27.4]	56	16.3 [6.9, 33.8]
No response		49	25.1 [8.7, 54.1]	22	2.8 [0.3, 20.9]	42	22.2 [9.3, 44.1]	34	14.3 [5.4, 32.7]
P-values			*0.169*		*0.398*		*0.401*		*0.797*
Infant currently on ARV prophylaxis									
No		37	15.2 [6.3, 32.5]	53	4.1 [1.2, 13.3]	104	15.0 [8.0, 26.2]	99	16.2 [8.2, 29.4]
Yes		90	15.1 [8.1, 26.5]	22	12.8 [3.1, 40.4]	18	26.1 [8.5, 57.5]	10	23.6 [4.0, 69.4]
P-values			*0.984*		*0.161*		*0.145*		*0.627*
Infant HIV status at enrolment									
Negative		74	17.5 [8.9, [illegible]]	61	8.6 [2.7, 24.3]	113	19.0 [10.1, 33.0]	97	13.3 [6.3, 25.9]
Positive		3	0.0	1	0.0	1	0.0	5	23.1 [3.0, 74.7]
Unknown		51	12.2 [5.8, 24.1]	13	0.0	9	0.0	8	37.3 [15.9, 65.3]
P-values			*0.473*		*[illegible]*		*0.330*		*0.930*
Gestational age at birth[a]									
≤37 weeks		17	20.6 [12.0, 33.1]	7	0.0	20	[illegible] [[illegible], 27.8]	23	31.1 [21.4, 42.8]
38 - 42 weeks		110	11.9 [7.3, 19.0]	67	8.4 [2.4, 25.5]	103	[illegible] [10.2, 36.3]	87	10.9 [3.9, 26.8]
P-values			*0.154*		*0.510*		*0.078*		*0.086*
Infant birth weight[a]									
Birth weight≥2.5kg		116	14.0 [7.2, 25.4]	71	6.4 [1.7, 21.0]	109	16.9 [10.3, 26.4]	96	15.8 [8.8, 26.9]
Low birth weight		12	26.2 [17.1,38.0]	3	16.7 [0.9, 81.9]	14	22.5 [3.7, 68.9]	14	20.3 [4.8, 56.0]
P-values			*0.062*		*0.499*		*0.59*		*0.68*

For peer review only

[a]denominator less than N due to missing responses; BMI – body mass index; ART antiretroviral therapy; ARV- antiretroviral; PMTCT – prevention of mother-to-child transmission of HIV; ANC – antenatal care; CI – confidence interval. P-values are from a chi-squared test. Significant p-values <0.05 are in boldface font.

For peer review only

For peer review only - http://bmjopen.bmj.com/site/about/guidelines.xhtml

BMJ Open

HIV viral load non-suppression and associated factors among pregnant and postpartum women in rural northeastern South Africa: a cross-sectional survey

Journal:	*BMJ Open*
Manuscript ID	bmjopen-2021-058347.R1
Article Type:	Original research
Date Submitted by the Author:	07-Feb-2022
Complete List of Authors:	Ngandu, Nobubelo; South African Medical Research Council<br>Lombard, Carl; University of Stellenbosch, Division of Epidemiology and Biostatistics<br>Mbira, Thandiwe ; South African Medical Research Council<br>Puren, Adrian; National Institute for Communicable Diseases; National Institute for Communicable Diseases<br>Waitt, Catriona; University of Liverpool Faculty of Health and Life Sciences, Department of Pharmacology<br>Prendergast, Andrew ; Queen Mary University of London<br>Tylleskär, Thorkild; Universitetet i Bergen, Centre for International health<br>Van de Perre, Philippe; INSERM UMR 1058, Pathogenesis and control of chronic infections; Université de Montpellier,<br>Goga, Ameena; South African Medical Research Council
**Primary Subject Heading**:	Epidemiology
Secondary Subject Heading:	Health services research, HIV/AIDS, Paediatrics
Keywords:	HIV & AIDS < INFECTIOUS DISEASES, Community child health < PAEDIATRICS, Epidemiology < INFECTIOUS DISEASES

SCHOLARONE™
Manuscripts

For peer review only - http://bmjopen.bmj.com/site/about/guidelines.xhtml

I, the Submitting Author has the right to grant and does grant on behalf of all authors of the Work (as defined in the below author licence), an exclusive licence and/or a non-exclusive licence for contributions from authors who are: i) UK Crown employees; ii) where BMJ has agreed a CC-BY licence shall apply, and/or iii) in accordance with the terms applicable for US Federal Government officers or employees acting as part of their official duties; on a worldwide, perpetual, irrevocable, royalty-free basis to BMJ Publishing Group Ltd ("BMJ") its licensees and where the relevant Journal is co-owned by BMJ to the co-owners of the Journal, to publish the Work in this journal and any other BMJ products and to exploit all rights, as set out in our licence.

The Submitting Author accepts and understands that any supply made under these terms is made by BMJ to the Submitting Author unless you are acting as an employee on behalf of your employer or a postgraduate student of an affiliated institution which is paying any applicable article publishing charge ("APC") for Open Access articles. Where the Submitting Author wishes to make the Work available on an Open Access basis (and intends to pay the relevant APC), the terms of reuse of such Open Access shall be governed by a Creative Commons licence – details of these licences and which Creative Commons licence will apply to this Work are set out in our licence referred to above.

Other than as permitted in any relevant BMJ Author's Self Archiving Policies, I confirm this Work has not been accepted for publication elsewhere, is not being considered for publication elsewhere and does not duplicate material already published. I confirm all authors consent to publication of this Work and authorise the granting of this licence.

For peer review only

HIV viral load non-suppression and associated factors among pregnant and postpartum women in rural northeastern South Africa: a cross-sectional survey

Nobubelo Kwanele Ngandu (corresponding author): HIV Prevention Research Unit, South African Medical Research Council, P. O. Box 19070, Tygerberg 7505, Cape Town, South Africa. Email: Nobubelo.Ngandu@mrc.ac.za

Carl J. Lombard: Biostatistics Unit, South African Medical Research Council, Cape Town, South Africa; Division of Epidemiology and Biostatistics, Department of Global Health, University of Stellenbosch, Cape Town, South Africa

Thandiwe Elsie Mbira: HIV Prevention Research Unit, South African Medical Research Council, Cape Town, South Africa

Adrian Puren: Centre for HIV and STI, National Institute of Communicable Diseases, Johannesburg, SA, South Africa.

Catriona Waitt: Department of Pharmacology, University of Liverpool, United Kingdom; Infectious Diseases Institute, Makerere University College of Health Sciences, Kampala, Uganda

Andrew J Prendergast: Blizard Institute, Queen Mary University of London, United Kingdom; Zvitambo Institute for Maternal and Child Health Research, Harare, Zimbabwe

Thorkild Tylleskar: Centre for International Health, University of Bergen, Norway

Philippe Van de Perre: Pathogenesis and control of chronic and emerging infections, University of Montpellier, INSERM, France; Etablissement Français du Sang, Antilles University, France; CHU Montpellier, Montpellier, France

Ameena Ebrahim Goga: HIV Prevention Research Unit, South African Medical Research Council, Cape Town, South Africa; Department of Pediatrics and Child Health, University of Pretoria, Pretoria, South Africa.

Word count = 4591

For peer review only - http://bmjopen.bmj.com/site/about/guidelines.xhtml

Abstract

Objectives: We aimed to measure the prevalence of maternal HIV viral load (mVL) non-suppression and assess associated factors, to evaluate progress towards UNAIDS targets.

Design: Cross-sectional study

Setting: The eight largest community health centers of Ehlanzeni, a rural district in northeast South Africa.

Participants: Pregnant women living with HIV (WLHIV) in their third trimester and postpartum WLHIV and their biological infants, recruited equally across all stages of the first 24 months postpartum, were included. A sample of 612 mothers participated from a target of 1000.

Primary outcome measures: The primary outcome was mVL non-suppression (defined here as mVL >1000 copies/mL). We collected information on antiretroviral use, healthcare visits and socio-demographics through interviews and measured plasma mVL. Descriptive statistics, chi-square tests and multivariable logistic regression analysis were conducted.

Results: All mothers (median age 30 years) were on antiretroviral therapy (ART) and 24.9% were on ART ≤12 months. The prevalence of mVL non-suppression was 14.7% (95% confidence interval (CI) 11.3%-19.0%), while 13.8% had low-level viraemia (50-1000 copies/mL). Most (68.9%) women had initiated breastfeeding and 37.6% were currently breastfeeding their infants. Being younger than 25 years (AOR 2.6[95%CI 1.1-6.4]), on first-line ART (adjusted odds ratio (AOR) 2.3[95%CI 1.1-4.6]) and married/cohabiting (AOR 1.9[95%CI 1.0-3.7]) were significantly associated with increased odds of mVL non-suppression.

Conclusions: The prevalence of mVL ≤1000 copies/mL of 85.3% among pregnant and postpartum WLHIV and attending public healthcare centers in this rural district is below the 2020 90-90-90 and 2030 95-95-95 UNAIDS targets. Given that low-level viraemia may also increase the risk of vertical HIV transmission, we recommend strengthened implementation of the new guidelines which include better ART options, improved ART regimen switching and mVL monitoring schedules, and intensified psychosocial support for younger women, while exploring district-level complementary interventions, to sustain viral loads below 50 copies/mL amongst all women.

Key words: Viral load non-suppression, pregnant, postpartum, vertical HIV transmission

For peer review only - http://bmjopen.bmj.com/site/about/guidelines.xhtml

Strengths and limitations

- This study provides mVL non-suppression data from a rural setting where research of this kind is limited.
- The mVL measurements were conducted using the gold standard whole blood plasma rather than dried blood spots despite the challenging remote settings.
- The postpartum inclusion criterion targeted mothers attending clinics with their biological children and hence could present a sample biased towards biological mothers with better clinic attendance and less representative of mothers who mostly assign child healthcare visits to other caregivers
- The sample sizes achieved were lower than planned and much lower for the postpartum stages which did not overlap with routine child vaccination schedules, but there was no statistically significant difference in the proportion of mVL non-suppression between the postpartum stages.

For peer review only

Background

Maternal HIV viral load (VL) during pregnancy and breastfeeding is an indicator of the risk of vertical transmission of HIV (MTCT). The HIV test-and-treat approach and universal coverage of daily life-long triple antiretroviral therapy (ART) to achieve viral suppression and prevent MTCT have contributed to substantial decreases in MTCT globally, although the incidence of vertical transmission remains a public health concern[1]. In South Africa, the national average MTCT rate has reduced to less than 2% at birth, but heterogeneity exists at sub-regional levels with much higher intrauterine infections in some districts[2]. In addition, the MTCT risk during the breastfeeding period is critical, because breast milk transmission globally is declining at a slower rate than in utero and intrapartum transmission[3]. Postpartum transmission is perpetuated by incident maternal HIV infection during the breastfeeding period combined with low use of repeat HIV testing amongst previously uninfected mothers; inadequate monitoring of maternal VL (mVL) and poor postpartum ART adherence[4-12]. Antenatal HIV prevalence has remained around 30% over the past decade in South Africa, and as high as 45% in some districts, making monitoring of mVL a necessity to ensure corrective measures in order to reduce MTCT risk[13].

In South Africa, although the target of ensuring MTCT below 5% percent at the end of breastfeeding was achieved by 2015, the case rate of less than 50 new HIV infections per 100 000 live births, to meet global elimination of MTCT (eMTCT) targets, has not been met[4 14]. Until the UNAIDS 95-95-95 goals are achieved and sustained across the PMTCT cascade from pregnancy through to the end of breastfeeding, meeting the eMTCT targets could be impossible[15 16]. The first two 95s, i.e., 95% of HIV-infected persons knowing their HIV status and 95% of these initiated on ART, are measured during the first antenatal care (ANC) visit and have been periodically reviewed to guide service delivery strengthening in South Africa[13 17]. In South Africa the UNAIDS 2020 targets of 90% are achieved for these two indicators at ANC entry level but, similar to other low- and middle-income countries (LMICs) sustaining adequate coverage of HIV diagnoses and ART adherence throughout the antenatal and postnatal period is challenging[15 18-20]. Considering that up to a third of pregnant women living with HIV (WLHIV) only initiate ART after enrolling into ANC, strengthening retention in care to support ART adherence and to monitor VL throughout the PMTCT cascade is critical[13]. Monitoring mVL needs to be a priority as it is the best indicator of ART efficacy, ART adherence, and need for enhanced infant postnatal prophylaxis in infants at high risk of HIV acquisition. Despite its importance, the last 95 (95% of all HIV-infected persons on ART should be virally suppressed, i.e., have VL below 1000 copies/mL) is not being monitored at a population level along the

For peer review only - http://bmjopen.bmj.com/site/about/guidelines.xhtml

PMTCT cascade in many LMICs with high HIV burden, including South Africa. This is largely attributed to the financial cost and logistical complications associated with VL measurements. Given the high heterogeneity at sub-regional levels, routine VL monitoring should be achievable by prioritizing hotspot districts currently bearing the highest antenatal and postnatal HIV burden and MTCT risk.

Study objective: We identified one of the hotspot districts with high maternal HIV prevalence and high MTCT in South Africa and conducted a cross-sectional evaluation of the prevalence of mVL non-suppression (VL>1000 copies/mL) and associated factors during peripartum and postpartum periods, to evaluate progress towards the UNAIDS targets.

For peer review only

Methods

Study design and inclusion criteria

A facility-based cross-sectional study was conducted at the eight largest community healthcare centers (CHC) in Ehlanzeni district, Mpumalanga Province, in rural northeastern South Africa (bordering Mozambique); seven were in rural localities and one in a peri-urban locality. A district-level sample size was calculated using an assumed mVL non-suppression prevalence of 25% and a precision of 5% at a 90% confidence level[6 21]. The sampling strategy was designed to equally represent different PMTCT cascade milestones across the peripartum and postpartum stages and grouped according to the 2015-2019 PMTCT guidelines for infant antiretroviral prophylaxis and breastfeeding[22]. The CHCs were treated as the primary sampling units for data collection. Therefore, the planned study design targeted equal sample sizes of N=200 (25 from each CHC) for each of five groups of WLHIV: third trimester of pregnancy, and four postpartum time points (0-14 weeks, 15-26 weeks, 27-52 weeks (6-11 months) and 53-104 weeks (12-24 months)). The inclusion criteria were: woman being in any of these stages, living with HIV and the baby present (for postpartum) on the day of interview. Data collection was conducted over a period of three months from mid-September to mid-December 2019 to minimize heterogeneity within the sample.

Data collection and laboratory measurements

The study was introduced to all female clinic attendees in waiting rooms and interested pregnant and postpartum women were screened for inclusion criteria after receiving their routine care. After written informed consent, the woman was interviewed to collect basic demographic and HIV-related clinical histories from recall and clinic records, and entered directly onto tablets linked to an electronic database

For peer review only - http://bmjopen.bmj.com/site/about/guidelines.xhtml

(REDCap) securely managed at the host institution[23]. Whole blood was collected from all women for VL measurement and transported to the local laboratory on ice. Plasma was separated within 4-12 hours and stored at -20 degrees Celsius until further analyses at the research laboratory. The HIV-1 viral load assays were performed using the Roche COBAS® AmpliPrep/COBAS® TaqMan® HIV-1 quantitative Test, version 2.0 (Roche Diagnostics GmbH, Mannheim, Germany), according to the manufacturer's instructions. The limit of virus detection using this assay was 20 copies/mL at minimum plasma volumes of 1-2 ml.

Independent variables

Sociodemographic factors: Maternal age and body mass index (BMI) were presented as categorical variables. A wide BMI range was observed and grouped into- underweight (13.0-18.4), normal (18.5-24.9), overweight (25.0-29.9), obese (30.0-39.9) and extremely obese (40.0-80.0), with extreme outliers treated as missing. Primary source of income was categorical and average household income was binary defined using the national poverty line household income cutoff of R3200/month. Marital status was either married/cohabiting or other (single, divorced, widow, undisclosed). The highest education achieved was either none, first 7 years of basic education (primary), years 8-12 (secondary), a post-secondary certificate (Tertiary-certificate) and post-secondary diploma or higher qualification (Tertiary- Diploma or higher). Participant self-reported partner's HIV status was positive, negative or unknown and frequency of condom use was never, sometimes or always.

ANC variables: Planned pregnancy (yes/no) was self-reported and gestation age at first ANC visit (≤12 weeks, 13-20 weeks, >20 weeks) and number of ANC visits (0-4 or 5-12 visits) were confirmed using clinic records for the most recent pregnancy.

ART-related factors: These included: duration on ART (≤12 months versus >12 months), current ART regimen (first-line regimen versus all others), adherence to ART defined as missed an ART dose during the most recent 7 days (yes/no), ever face challenges with ART adherence (yes/no), and timing of HIV-positive diagnosis (before most recent pregnancy versus during ANC/after). The recommended ART regimens at the time of this study were a fixed-dose combination of TDF+3TC (or FTC) +EFV with AZT as an option in the case of a contra-indication for first-line and TDF(or AZT) +3TC(or FTC) +LPV/r(or ATV/r) for second line options[22]. Switch to second line was expected after two consecutive VL>1000 copies/mL 6 months apart on first-line ART or after two months of intensive adherence counselling and VL remaining >1000 copies/mL. Switch to third line regimen was recommended after a VL>1000 was sustained for over 6 months on second line and the drug options were determined through specialist review of drug resistant mutations. All women were tested for HIV at ANC first visit (ANC-1) and every 6 months thereafter [22].

For peer review only - http://bmjopen.bmj.com/site/about/guidelines.xhtml

Infant-related postnatal factors: Infant age was used to design the study strata. Child health booklets and maternal report were used to obtain infant HIV status at enrolment (positive, negative, unknown), gestational age at birth (≤37 weeks or 38-42 weeks) and birth weight (low birth weight (<2.5kg) versus higher). Mothers self-reported whether infant was currently breastfeeding (yes/no), ever breastfed (yes/no, no response) and currently on anti-HIV prophylaxis (yes/no).

Dependent variable

The primary outcome measure was mVL non-suppression (VL>1000 copies/mL), in line with the South African PMTCT guideline criteria for ART regimen management at the time of data collection[22].
The national PMTCT guidelines practiced during this study recommended 6-monthly VL testing if a woman is virally suppressed (VL≤1000 copies/mL) or 4-6 weekly if their viral load is >1000 copies/mL[22].

Statistical analyses

We conducted univariable and multivariable logistic regression analyses to measure the association between the outcome and exposures of interest. Exposure variables were included in the multivariable model only if they had an overall univariable p-value <0.2. A significance level of 0.05 in the multivariable analysis was used to indicate a significant association with mVL. Infant-related PMTCT postnatal factors were evaluated for association with mVL separately. Chi-square tests were used to assess associations between categorical variables and descriptive statistics were done.
In all tests conducted, a stratified survey-based analysis was used to combine the five study groups into a single analysis. As per study design, the study groups were specified as the strata and the CHCs were treated as primary sampling units, in the survey structure for data analysis. In addition, all proportions and odds ratios were adjusted (weighted) for sample size attained by specifying survey weights in the survey structure and in all analysis. The survey weights were calculated by taking the inverse of sample-size realization proportion, where proportions were calculated using the primary sampling unit (facility-level) sample target of N=25 within each study group stratum. Study group stratification was not applied in the supplementary descriptive analyses of each study group separately.
The STROBE checklist for observational cross-sectional studies was followed (**Supplementary File1**).

For peer review only - http://bmjopen.bmj.com/site/about/guidelines.xhtml

Ethics approval was granted by the South African Medical Research Council ethics committee (EC002-2-2019) and the Regional Committee for Medical and Health Research Ethics West, Norway (REK-Vest no 2019/773).

Patient and public involvement: No patient involved.

Results

During the 3-month data collection period, interview data were successfully collected from 667 WLHIV, translating to sample size realization of: 66% overall – within group ascertainment was 88% for third trimester group (n=176), 64% for 0-14 weeks postpartum group (n=128), 38% for 15-26 weeks postpartum group (n=75), 62% for 27-52 weeks postpartum group (n=123) and 55% for 53-104 weeks postpartum group (n=110). The precision for these achieved samples remained close to 0.05 and was 0.05, 0.0514, 0.068, 0.0526 and 0.056, respectively. Very few mother-infant pairs in the 15-26 weeks postpartum group, a period with no routine vaccination schedules, were present in the clinics. VL results were available for 612/667 (91.8%) interviewed women; 55 (8.2%) samples had insufficient plasma. The 55 women with insufficient plasma were excluded from the analysis.

Description of the study population

Socio-demogaphics and ANC: The median age was 30 years (interquartile range 26-35), 8.6% and 58.1% had 0-7 years and 8-12 years of completed education, respectively, while the remaining third had some tertiary education. Most (59.6%) came from households with average earnings below the poverty income line (<R3200 per month) (**Table 1**). A quarter of the participants were employed, a third relied on government grants while the remainder depended on another individual (parent/relative/spouse/partner) as their main source of income; 43.5% women were married or cohabiting; 46.4% knew they were in a concordant HIV-positive sexual relationship while 35.4% did not know their partner's HIV status. Half of the participants had an unplanned pregnancy and around two thirds had their first ANC visit during the first twelve weeks of gestation and achieved at least 5 ANC visits.

For peer review only - http://bmjopen.bmj.com/site/about/guidelines.xhtml

ART-related factors: Over 70% of participants were diagnosed with HIV before the current/recent pregnancy. All 612 women were already on ART at study enrolment and three quarters had been on ART for more than 12 months. Most (80.2%) women were still on their first-line regimen. Although self-reported levels of adherence to ART during the most recent 7 days were very high (95.2%), 38.0% reported ever experiencing challenges which potentially interfere with adherence to ART. The commonly cited barriers were time inconvenience (22.8%), non-disclosure of HIV status (11.7%), ART side effects (5.4%) and lack of support from family/partner (3.2%).

Infant-related postnatal factors: Close to 70% of the infants were ever breastfed and 37.6% were currently breastfeeding. Overall, 29% infants were still taking prophylaxis, higher than the 0-14 weeks postnatal sample proportion of 23% expected to be on prophylaxis. At enrolment, 79.2% of mothers knew their infants to be HIV-negative and 2.1% to be HIV-positive while 18.7% did not know the infant's HIV status. Over 80% of infants were born full-term and with weight ≥2.5 kilograms.

Table 1: Summary of study population characteristics

	n	%
All	612	100
**Study group**		
3rd trimester	176	28.8
0-14 weeks postpartum	128	20.9
15-26 weeks postpartum	75	12.2
27-52 weeks postpartum	123	20.1
53-104 weeks postpartum	110	18.0
**Socio-demographics and ANC**		
Age in years		
15 – 24	105	17.8
25 - 34	351	57.2
35 - 46	156	25.0
BMI[a]		
13.0 - 18.4	20	3.6
18.5 - 24.4	200	35.4
25.0 - 29.9	175	28.6
30.0 - 39.9	173	27.1
40.0 - 80.0	39	5.3
Education		
None	8	1.2
Primary (1-7 years)	44	7.4

For peer review only - http://bmjopen.bmj.com/site/about/guidelines.xhtml

Secondary (8-12 years)	347	58.1
Tertiary- certificate	155	23.3
Tertiary- Diploma or higher	58	10.0
Married/Cohabiting		
No	362	56.5
Yes	250	43.5
Income source[a]		
Employed	163	24.2
Spouse/partner	187	32.5
Parent/relative	90	14.2
Grant	166	29.1
Household gross income/month		
>R3200	262	40.4
R3200 or less/none	349	59.6
Partner's HIV status[a]		
Negative	110	18.1
Positive	279	46.4
Don't know	222	35.4
Condom use frequency[a]		
Never	52	7.2
Sometimes	226	35.8
Always	330	57.0
Planned pregnancy		
No	330	52.3
Yes	282	47.7
Gestation at ANC-1 visit		
≤12 weeks	379	65.8
13-20 weeks	165	23.9
>20 weeks	68	10.3
Number of ANC visits[a]		
0-4 visits	210	32.2
5-12 visits	401	67.8
**PMTCT exposure variables**		
Timing of HIV-positive result		
Before pregnancy	448	73.1
At ANC-1 or after	164	26.9
Time since ART initiation		
>12 months	442	85.1
≤12 months	170	24.9
Current ART regimen[a]		
First line	491	80.2

For peer review only - http://bmjopen.bmj.com/site/about/guidelines.xhtml

2nd/3rd line or unknown	119	19.8
Missed an ART dose last 7 days		
No	582	95.2
Yes	30	4.8
Facing any ART adherence challenges		
No	376	62.0
Yes	236	38.0
**Infant related factors (postpartum sample only)**	**436**	**100**
Infant ever breastfed[a]		
No	146	31.1
Yes	289	68.9
Infant currently breastfeeding		
Yes	163	37.6
No	126	31.0
Chose not to disclose	147	31.4
Infant currently on ARV prophylaxis[a]		
No	293	71.0
Yes	140	29.0
Infant HIV status at enrolment		
Negative	345	79.2
Positive	10	2.1
Unknown	81	18.7
Gestational age at birth[a]		
≤37 weeks	67	18.7
38 - 42 weeks	367	81.3
Infant birth weight[a]		
Birth weight ≥2.5kg	392	90.6
Low birth weight (<2.5 kg)	43	9.4

[a]denominator less than N due to missing responses; ANC- antenatal care; PMTCT – prevention of mother-to-child transmission of HIV; ART – antiretroviral therapy; ARV – antiretroviral; BMI – body mass index; ART - antiretroviral therapy.

Prevalence of maternal viral load non-suppression

Maternal viral load ranged between undetectable and 557 500 copies/mL. The median of detectable values was 138 copies/mL (interquartile range 35-3660 copies/mL). Overall, 14.7% [95% confidence interval (CI): 11.3-19.0] of the women had mVL non-suppression while 13.8% [95% CI: 10.2-18.4] had low-level viraemia (VL 50-1000 copies/mL) and 71.5% [95% CI: 63.7-78.2] had policy-defined undetectable

For peer review only - http://bmjopen.bmj.com/site/about/guidelines.xhtml

(<50 copies/mL) mVL[22]. VL non-suppression by study group ranged between 6.9% and 17.5%, with all groups except the 15-26 weeks postpartum group, showing a prevalence of 15.0% or higher (**Table 2**). Although mVL non-suppression was lower for the participants in the 15-26 weeks group, there was no statistically significant difference across PMTCT stages (chi-squared p-value (p)=0.284).

Summary of study population characteristics by maternal viral load non-suppression

The prevalence of mVL non-suppression differed significantly by source of income (p=0.018), ART duration (p=0.029) and ART regimen (p=0.016) (**Table 2**). The proportion of VL>1000 copies/mL was higher amongst women who depended on a parent/relative or spouse/partner as their main source of income, were on ART for ≤12 months, or were on first-line ART.

Weak evidence of differences in the prevalence of mVL non-suppression were also observed when women were grouped by maternal age (p=0.054), BMI (p=0.075), household income level (p=0.097), knowledge of partner's HIV status (p=0.081) and timing of first HIV-positive result (p=0.086). The proportion of viral non-suppression tended to be higher amongst women who were: younger than 25 years of age, had BMI between 18.5-29.9, came from households with average income >R3200, aware that their partner was HIV-positive or did not know their partner's HIV status or received the first HIV-positive diagnoses after enrolling into ANC for the most recent pregnancy.

Infant related postnatal PMTCT characteristics did not appear to distinguish women by prevalence of viral load non-suppression (**Table 2**).

The distribution of mVL non-suppression by participant charateristics within each study group separately is presented in the supplementary data (**Supplementary File2**).

Table 2: Summary of study population characteristics by maternal viral load non-suppression

		VL≤ 1000 copies/mL		VL> 1000 copies/mL		p-value
		n	% [95% CI]	n	% [95% CI]	
**All**		526	85.3 [81.6, 88.3]	86	14.7 [11.7, 18.4]	

For peer review only - http://bmjopen.bmj.com/site/about/guidelines.xhtml

Study group	3rd trimester	147	82.9 [76.9, 87.6]	29	17.1 [12.4, 23.1]	*0.284*
	0 - 14 weeks postpartum	112	85.0 [76.5, 90.8]	16	15.0 [9.2, 23.5]	
	15 - 26 weeks postpartum	70	93.1 [82.5, 97.4]	5	6.9 [2.6, 17.5]	
	27 - 52 weeks postpartum	102	82.5 [71.1, 90.1]	21	17.5 [10.0, 28.9]	
	53 -104 weeks postpartum	95	83.6 [74.5, 89.9]	15	16.4 [10.1, 25.2]	
**Maternal socio-demographics and ANC**						
Age in years	15 – 24	82	76.5 [65.5, 84.8]	23	23.5 [15.2, 34.5]	*0.054*
	25 - 34	305	86.2 [80.3, 90.5]	46	13.8 [9.5, 19.7]	
	35 - 46	139	89.5 [82.8, 93.8]	17	10.5 [6.2, 17.2]	
BMI	13.0 - 18.4	17	88.1 [63.0, 97.0]	3	11.9 [3.0, 37.0]	*0.075*
	18.5 - 24.9	171	84.1 [76.5, 89.6]	29	15.9 [10.4, 23.5]	
	25.0 - 29.9	140	79.4 [70.1, 86.4]	35	20.6 [13.6, 29.9]	
	30.0 - 39.9	157	90.7 [86.6, 93.7]	16	9.3 [6.3, 13.4]	
	40.0 - 80.0	36	92.5 [82.6, 97.0]	3	7.5 [3.0, 17.4]	
Education	None	6	66.5 [31.0, 90.0]	2	33.5 [10.3, 69.0]	*0.333*
	Primary (1-7 years)	41	94.5 [81.2, 98.5]	3	5.5 [1.5, 18.8]	
	Secondary (8-12 years)	297	84.0 [78.5, 88.4]	50	16.0 [11.6, 21.5]	
	Tertiary- certificate	131	85.8 [78.6, 90.9]	24	14.2 [9.1, 21.4]	
	Tertiary- Diploma/higher	51	86.7 [70.5, 94.7]	7	13.3 [5.3, 29.5]	
Married/Cohabiting	No	319	87.7 [83.8, 90.9]	43	12.3 [9.1, 16.3]	*0.122*
	Yes	207	82.1 [74.7, 87.7]	43	17.9 [12.3, 25.3]	
Income source[a]	Employed	145	88.9 [80.6, 94.0]	18	11.1 [6.0, 19.4]	***0.018***
	Spouse/partner	153	80.9 [72.6, 87.2]	34	19.1 [12.8, 27.4]	
	Parent/relative	72	75.9 [62.8, 85.4]	18	24.1 [14.6, 37.2]	
	Grant	150	91.3 [85.5, 94.9]	16	8.7 [5.1, 14.5]	
Household monthly gross income	>R3200	215	82.0 [75.9, 86.8]	47	18.0 [13.2, 24.1]	*0.097*
	R3200 or less/none	310	87.5 [82.8, 91.1]	39	12.5 [9.0, 17.2]	
Partner's HIV status	Negative	98	91.6 [85.8, 95.2]	12	8.4 [4.8, 14.2]	*0.081*
	Positive	243	85.0 [79.1, 89.4]	36	15.0 [10.6, 20.9]	
	Don't know	184	82.4 [76.4, 87.2]	38	17.6 [12.8, 23.6]	
Condom use frequency[a]	Never	43	85.9 [75.4, 92.3]	9	14.1 [7.7, 24.6]	*0.235*
	Sometimes	200	88.6 [82.6, 92.8]	26	11.4 [7.2, 17.4]	
	Always	280	83.2 [77.5, 87.6]	50	16.9 [12.4, 22.5]	
Planned pregnancy	No	289	86.2 [81.0, 90.2]	41	13.8 [9.8, 19.0]	*0.611*
	Yes	237	84.3 [77.9, 89.1]	45	15.7 [10.9, 22.1]	
Gestational age at ANC-1 visit	≤12 weeks	335	86.6 [81.8, 90.3]	44	13.4 [9.7, 18.2]	*0.443*
	13-20 weeks	136	83.2 [76.2, 88.5]	29	16.8 [11.6, 23.8]	
	>20 weeks	55	81.9 [70.9, 89.4]	13	18.1 [10.6, 29.1]	
Number of ANC visits[a]	0-4 visits	180	85.1 [79.5, 89.3]	30	14.9 [10.7, 20.5]	*0.921*
	5-12 visits	345	85.4 [81.1, 88.8]	56	14.6 [11.2, 18.9]	
**ART-related factors**						

For peer review only - http://bmjopen.bmj.com/site/about/guidelines.xhtml

Timing of HIV-positive result	Before pregnancy	394	87.3 [82.6, 90.9]	54	12.7 [9.1, 17.4]	*0.086*
	At ANC-1 or after	132	79.8 [71.1, 86.4]	32	20.2 [13.6, 28.9]	
Time since ART initiation	>12 months	392	87.5 [83.0, 90.9]	50	12.5 [9.1, 17.0]	***0.029***
	≤12 months	134	78.6 [70.3, 85.0]	36	21.4 [15.0, 29.7]	
Current ART regimen[a]	2nd/3rd line or unknown	107	92.3 [85.9, 95.9]	12	7.7 [4.1, 14.2]	***0.016***
	First-line	417	83.5 [79.5, 86.9]	74	16.5 [13.1, 20.5]	
Missed an ART dose last 7 days	No	501	85.7 [82.1, 88.6]	81	14.4 [11.4, 17.9]	*0.294*
	Yes	25	78.1 [56.0, 90.9]	5	21.9 [9.1, 44.1]	
Facing any ART adherence challenges	No	331	86.3 [81.3, 90.0]	45	13.8 [10.0, 18.7]	*0.551*
	Yes	195	83.7 [75.8, 89.4]	41	16.3 [10.6, 24.2]	
**Infant related factors (postpartum sample only)**						
	All	379	85.8 [81.4, 89.4]	57	14.2 [10.6, 18.6]	
Infant HIV status at enrolment	Negative	296	85.4 [80.3, 89.4]	49	14.6 [10.6, 19.7]	*0.882*
	Positive	9	88.3 [50.1, 98.3]	1	11.7 [1.7, 49.9]	
	Unknown	74	87.5 [72.6, 94.9]	7	12.5 [5.1, 27.5]	
Infant currently on ARV prophylaxis[a]	No	258	87.0 [81.8, 90.0]	35	13.0 [0.1, 18.2]	*0.326*
	Yes	119	83.5 [75.3, 89.4]	21	16.5 [10.7, 24.7]	
Infant ever breastfed[a]	No	124	82.3 [72.7, 89.1]	22	17.8 [11.0, 27.3]	*0.231*
	Yes	254	87.4 [82.5, 91.0]	35	12.6 [9.0, 17.5]	
Infant currently breastfeeding	Yes	143	87.5 [81.4, 91.8]	20	12.5 [8.2, 18.6]	*0.427*
	No	111	87.2 [79.9, 92.1]	15	12.8 [7.9, 20.1]	
	Chose not to disclose	125	82.5 [73.0, 89.2]	22	17.5 [10.8, 27.0]	
Gestational age at birth[a]	≤37 weeks	56	83.1 [70.9, 90.8]	11	16.9 [9.2, 29.1]	*0.466*
	38 - 42 weeks	322	86.9 [81.9, 90.7]	45	13.1 [9.3, 18.1]	
Infant birth weight[a]	Birth weight≥2.5kg	345	86.6 [82.3, 89.9]	47	13.4 [10.1, 17.7]	*0.151*
	Low birth weight	33	78.4 [61.9, 89.1]	10	21.6 [10.9, 38.1]	

[a]denominator less than N due to missing responses; ANC-1 – antenatal care first visit; ARV – antiretroviral; BMI – body mass index; ART antiretroviral therapy; CI – confidence interval, VL – viral load. P-values are from a chi-squared test. Significant p-values <0.05 are in boldface font.

Factors associated with maternal viral load non-suppression

In univariable analyses to identify factors associated with mVL non-suppression amongst all women, independent variables with an overall odds ratio (OR) p-value<0.2 and included in the multivariable logistic regression model were: ART duration, ART regimen maternal age, BMI, marital status, household income level, partner's HIV status and condom use (**Table 3**). The 'timing of first HIV positive result'

For peer review only - http://bmjopen.bmj.com/site/about/guidelines.xhtml

variable was excluded from the multivariable model because it showed collinearity with ART duration (Variance Inflation factor of the interaction term was above 5, i.e., 6.3). The OR p-values of other variables including that for the study group stratification were >0.2 and hence excluded from the adjusted model. All infant-related postnatal variables had OR p-values >0.2 and an adjusted model analyses was not conducted (**Table 4**).

In the adjusted model (**Table 3**), the odds of viral non-suppression were significantly increased amongst women who were younger than 25 years compared to those aged 35-46 years (adjusted odds ratio (AOR) 2.6 [95% CI: 1.1-6.4], p=0.037), were on first-line ART regimen (AOR 2.3 [95% CI: 1.1-4.6], p=0.026) and were married or cohabiting (AOR 1.9 [95% CI: 1.0-3.7], p=0.042). Weak associations were observed for increased odds of viral non-suppression amongst women who initiated ART within the most recent 12 months (AOR 1.7 [95% CI: 0.8-3.6], p=0.126) or did not know their male partner's HIV status (AOR 2.1 [95% CI: 0.9-4.8], p=0.080).

Extremely obese BMI of ≥40 was significantly associated with reduced odds of viral non-suppression compared to normal BMI of 18.5-24.9 (AOR 0.3 [95% CI: 0.1-0.9], p=0.028). There was no difference between normal BMI and either underweight, overweight or obese BMI.

Table 3: Factors associated with maternal viral load non-suppression among all women

	**OR [95% CI]**	**p-value**	**AOR [95% CI]**	**p-value**
Time since ART initiation				
>12 months	ref			
≤12 months	1.9 [1.1, 3.4]	**0.031**	1.7 [0.8,3.6]	0.126
Current ART regimen				
2nd/3rd line or unknown	ref			
First-line	2.4 [1.2, 4.8]	**0.018**	2.3 [1.1,4.6]	**0.026**
Age in years				
35 - 46	ref			
25 - 34	1.4 [0.7,2.8]	0.376	1.1 [0.5,2.4]	**0.733**
15 - 24	2.6 [1.1,6.2]	**0.029**	2.6 [1.1,6.4]	**0.037**
BMI				
18.5 - 24.9	ref			
13.0 - 18.4	0.7 [0.1, 3.8]	0.689	0.8 [0.2,4.4]	0.8146
25.0 - 29.9	1.4 [0.7,2.8]	0.379	1.4 [0.6,2.9]	0.415
30.0 - 39.9	0.5 [0.3,1.0]	0.051	0.5 [0.3,1.1]	0.089
40.0 - 80.0	0.4 [0.2,1.2]	0.112	0.3 [0.1,0.9]	**0.028**

For peer review only - http://bmjopen.bmj.com/site/about/guidelines.xhtml

Married/Cohabiting				
No	ref			
Yes	1.6 [0.8, 2.8]	0.124	1.9 [1.0,3.7]	**0.042**
Partner's HIV status				
Negative	ref			
Positive	1.9 [0.9,4.3]	0.101	1.9 [0.9,4.1]	0.096
Don't know	2.3 [1.2,4.7]	**0.020**	2.1 [0.9,4.8]	0.080
Condom use frequency				
Never	ref			
Sometimes	0.8 [0.3,1.9]	0.579	0.8 [0.3,2.0]	0.628
Always	1.2 [0.6,2.6]	0.583	1.1 [0.6,2.1]	0.769
Household gross income/month				
>R3200	ref			
R3200 or less/none	0.6 [0.3, 1.1]	0.099	0.6 [0.4,1.0]	0.073

ANC-1 – antenatal care first visit; BMI – body mass index; ART- antiretroviral therapy; OR – Odds ratio; AOR – adjusted odds ratio; CI – confidence interval. Significant p-values <0.05 are in boldface font.

For peer review only

Table 4: Association between infant related postnatal factors and maternal viral load non-suppression

	**OR [95% CI]**	**p-value**
All		
Gestational age at birth		
≤37 weeks	ref	
38 - 42 weeks	0.7 [0.3, 1.7]	0.467
Infant birth weight		
Birth weight≥2.5kg	ref	
Low birth weight	1.8 [0.8, 4.0]	0.156
Infant HIV status at enrolment		
Negative	ref	
Positive	0.8 [0.1, 5.8]	0.797
Unknown	0.8 [0.3, 2.4]	0.736
Infant currently on ARV prophylaxis		
No	ref	
Yes	1.3 [0.4, 2.4]	0.327
Infant ever breastfed		
No	ref	
Yes	0.7 [0.3, 1.3]	0.233
Infant currently breastfeeding		
Yes	ref	
No	1.0 [0.5, 2.0]	0.935
Chose not to disclose	1.4 [0.7, 3.0]	0.265

For peer review only - http://bmjopen.bmj.com/site/about/guidelines.xhtml

ARV - antiretroviral; OR – Odds ratio; CI – confidence interval.

Discussion

The prevalence of mVL<1000 copies/mL in this rural South African district was estimated at 85.3%, and is still below the UNAIDS 2020 and 2030 targets of 90% and 95%, respectively, amongst persons on ART[15 16]. The observed VL suppression prevalence is higher than the 2017 South Africa national antenatal survey estimate of 79.5%, indicating that the district is comparatively performing well[24]. The postpartum study inclusion criteria of biological mothers could be biasing results towards women who frequently attend healthcare services with their biological children, and possibly under-estimating mVL non-suppression prevalence without those women who assign child postnatal care activities to other caregivers. The observed prevalence is comparable to PMTCT population in other African countries such as Malawi (84-88%), but is lower than that reported in an urban setting (91%) within South Africa, further supporting the existing inter-district disparities in PMTCT performance in-country[6 19 25]. These sub-national variations highlight the need for district-level surveys as opposed to provincial-level surveys which mask existing heterogeneity at district-level.

Even though the cross-sectional study design limited our understanding of causality, the significantly increased odds of having unsuppressed VL among women on first-line ART regimens could be related to the observed high proportion of unsuppressed VL amongst women who were on ART for ≤12 months. Alternatively, it could be due to the dominance of efavirenz-based first-line regimen which has been shown to be associated with delayed viral suppression in this population[26 27] or a high population-level risk of drug resistance previously observed in South African rural populations[28], or a combination of these factors. The new guidelines currently being rolled-out offer pregnant women a dolutegravir-based regimen as a first-line choice and alternative to efavirenz regimen as part of the new efforts to improve viral load suppression[29]. The extremely high self-reported ART well-adherent (95.2%) group likely includes a desirability bias, but other studies in African settings have shown reliability of self-reported ART adherence including its association with mVL[25 30]. Therefore, we conclude that the disproportionately high prevalence of unsuppressed mVL in the group on first-line ART regimen implies a combination of challenges with adherence amongst those recently initiated on ART and first-line regimen failure. Findings

For peer review only - http://bmjopen.bmj.com/site/about/guidelines.xhtml

from Zimbabwe showed that being on second-line ART regimen versus first-line was associated with higher odds of viral suppression[31]. It is possible that regimen intervention or shift to second-line ART was delayed amongst some women. This is further supported by that three quarters of the study sample had been on ART for more than a year, and 90% had their first ANC visit early before 20 weeks gestation at which point over 70% already knew their HIV-positive status and were on ART.

One likely underlying driver of high mVL association with first-line ART is the 6-months long window recommended for following up those with low-level viraemia (50-1000 copies/mL), who however, are at risk of higher VL during this period. The 6-months window of routine follow-up visit when VL≤1000 copies/mL, was too long considering that it included low-level viraemia which may account for over 40% of early MTCT in some settings[3 6 22 25]. Given the new policies emphasizing targeting complete viral suppression of <50 copies/mL as opposed to VL<1000copies/mL, our results of 71.5% complete viral suppression shows that more work remains to be done[29]. The newly revised 2019 South African PMTCT guidelines have shortened the 6-months window to 8-10 weeks when VL is 50-999 copies/mL to address the problem of delayed VL and ART management, and have provided tips for the healthcare worker to understand barriers to ART adherence such as non-disclosure along with patient care interventions[29]. In addition, those newly initiated on ART are reviewed at 3 months regardless of baseline VL level, after which they are followed according to the universal schedule. These introduced shorter follow-up windows are expected to improve VL management going forward, particularly for those with recent ART initiation.

Periodic monitoring, evaluation and strengthening of these new guidelines as well as any outstanding service-delivery or user-related barriers is imperative. Client-related barriers and long turnaround times for availability of VL results are examples of long-standing challenges that interfere with retention in care and timely follow-up management of VL and ART in LMIC settings[7 32-35]. In a 2018 evaluation of this district and five others, for example, we found that on average at district-level, between 37-100% of clinics delay (>7 days) returning VL results to PMTCT clients after the clinic has received them from the laboratory[36]. Given that the most cited potential barrier to ART adherence in this current study was time inconvenience, we hypothesise that this factor contributed to women delaying clinic attendance for their follow-up visits. At present the country is implementing an electronic monitoring system called E-labs to shorten the turnaround times along the VL processing pipeline from blood draw at clinics until availability of results at the testing clinic[37]. Mobile phone messaging directly to patients, such as MomConnect, should be incorporated into E-labs to shorten time to clinic-patient contact[38]. Point of

For peer review only - http://bmjopen.bmj.com/site/about/guidelines.xhtml

care (POC) VL testing which is not common in this district should also be considered, given strong evidence for improved turnaround times when using POCs[39-41]. Alternatively, long-acting injectable antiretroviral therapeutics could ultimately reduce the burden of frequent clinic visits and daily pill adherence challenges, and thus studies developing these need to be strengthened earnestly[11].

The observed association of mVL non-suppression with young women aged 15-24 years is not unexpected. Earlier (2010-2014) studies, including a national PMTCT survey secondary analysis showed that adolescent PMTCT clients in South Africa are at higher risk of undesirable clinical and healthcare uptake outcomes[42 43]. Findings remain similar in the more recent (2016 - 2017) national representative data in South Africa where younger age was significantly associated with failure to achieve undetectable VL[24 44]. The revised PMTCT guidelines have included a guidance for adolescent-aware recommendations during antenatal and postpartum care. The guidance advises healthcare workers to identify psychosocial stressors and provide referrals for professional interventions, and emphasizes intensive adherence support such as enrolment into peer-led support groups[29]. Based on a systematic review of impactful interventions for improving healthcare uptake and adherence, family-centered initiatives could also be investigated for improving HIV health amongst pregnant and breastfeeding adolescents in this setting[45].

The high prevalence of unsuppressed VL observed amongst women who depended on another person including a spouse/partner for income aligns with the significantly increased odds amongst those who were married or cohabiting. The particularly high proportion (35%) of women who do not know their male partner's HIV status is also concerning and could be another underlying contributing factor. HIV disclosure to sexual partners, partner relations and partner involvement as determinants of utilization of HIV services and HIV health outcomes amongst pregnant and postnatal women have been long-time challenges in African settings[46-49]. Non-disclosure of HIV status was the second highest cited potential barrier to ART adherence (11.7%) in this study. Data from pregnant women from the same province as this study district, collected seven years ago, indicated non-disclosure of HIV status as a key factor in poor ART adherence[50]. Disclosure to a partner has been associated with better HIV care outcomes in mothers and their babies in Rwanda[51]. An earlier study in Zimbabwe observed reduced uptake of HIV care among eligible PMTCT clients living with a male partner[49]. Given challenges involving male partners and particularly for younger women, the adolescent-aware recommendations in the current PMTCT guidelines do emphasize on sexual reproductive health counselling during ANC and identification of socio-contextual

For peer review only - http://bmjopen.bmj.com/site/about/guidelines.xhtml

barriers such as abusive relationships to offer counselling referrals and ensure a safe and healthy home environment[29]. More work is however needed in this area.

The significant association between extreme BMI and reduced odds of VL non-suppression is not well understood and needs further investigation. A previous HIV vaccine efficacy trial reported an association between overweight/obese BMI and suppressed vaccine-induced immune response against HIV in a South African population[52]. Other separate studies on HIV-positive persons from different countries, initiated on antiretroviral treatment, found high BMI to be associated with better CD4 count recovery[53-55]. Whether our findings are simply due to the increased prevalence of obesity in South Africa (with women ranking in the highest burdened countries), and/or increased weight gain due to antiretrovirals as previously implied or there is an underlying biological mechanism, is unclear and needs further investigation[56-58].

Limitations

Although the 3-month recruitment period was expected to achieve the desired sample size, assuming 50% participation rate, the realized sample sizes were not as high as planned. However, there was no statistically significant heterogeneity in the primary outcome between the study groups, making the survey-weighted results reliable. The precision achieved by the realized sample size remained acceptable. The reduced sample size in the postpartum groups could have resulted from the study's inclusion criterion of biological mother-infant pairs. It is common practice locally to assign child healthcare visits to other caregivers. The 15-26 weeks postpartum study group had the lowest sample size which we attribute to lack of routine childhood vaccination points during this child growth period. The study by recruiting in facilities also excluded clients who had defaulted from care. Although the findings of our sample could have introduced a selection bias, the viral load suppression estimates lie within the range of other estimates reported in the country. Desirability bias from self-reported adherence to ART and condom use and knowing of male partner's HIV status, cannot be ruled out completely. Use of a 7-day recall for adherence to ART, although limiting recall bias, might suffer from low representativeness of the entire period since the last clinic contact. The possible influence of specific ART drugs could not be assessed due to lack of data on specific drug combinations used. This, however, may have minor effects given that options for the first-line regimen were limited to efavirenz-based regimens, hence not much diversity in specific drugs used [22].

For peer review only - http://bmjopen.bmj.com/site/about/guidelines.xhtml

For peer review only

The cross-sectional study design introduces inherent limitations. Causality could not be inferred in this cross-sectional study design and a prospective cohort approach would be needed to confirm some of the associations. An example of the limitation of a cross-sectional design in this context is not being able to understand the timing of infant HIV testing and the relationship between infant HIV-positivity versus mVL given there is no information on duration of non-suppression in the mother against attrition in care, interruptions in ART intake and feeding practices.

The sample was designed to measure prevalence of VL>1000 copies/mL and the data were not sufficient to investigate differences between VL stages within the 50-1000 copies/mL range to fully inform the newly implemented guidelines. The result of VL=50-1000 copies/mL is only reported descriptively. Virological failure and treatment failure could not be confirmed with certainty given only one VL measurement done.

Conclusions

The UNAIDS 2020/2030 targets for viral suppression amongst PMTCT clients in this district have not been achieved. This study highlights that socio-demographic factors (maternal age, BMI and marital/cohabiting status) and type of ART regimen disproportionately influence maternal VL suppression. While the role of BMI requires further investigation, recommendations outlined in the current South African PMTCT guidelines have potential to mitigate other identified factors through, amongst other revisions, the improved schedules for monitoring VL and ART regimen switching, better alternatives for ART drugs and targeted psycho-socio support for younger women with consideration of marital/male partner influence. Given that low-level viraemia may also increase the risk of vertical HIV transmission, it is imperative to periodically monitor, evaluate and strengthen the implementation of these guidelines alongside complementary district-level interventions, to keep all women virally suppressed.

Declarations

Acknowledgements: Authors would like to thank: the Ehlanzeni district PMTCT leadership and partners for supporting the placement of the study at the clinics; the Ehlanzeni local Rob Ferreira laboratory for handling of blood samples; the staff at NICD for conducting laboratory assays for viral load measurement;

For peer review only - http://bmjopen.bmj.com/site/about/guidelines.xhtml

the SAMRC data collection and coordinating team; and Dr Witness Chirinda for involvement in study planning discussions and contributing to data cleaning.

Availability of data and materials: The dataset supporting the conclusions of this article will be available after the primary objectives for the study have been published. Interested researchers should contact the corresponding author to confirm if data have become open access.

Competing interests: The authors declare that they have no financial or personal relationship(s) that may have inappropriately influences them in writing this article.

Funding & disclaimer: This study was funded by the National Department of Health – South Africa. Authors NKN & AEG were supported by the South African Medical Research Council. Author TEM was supported by the National Research Foundation – South Africa. AJP is funded by Wellcome (grant 108065/Z/15/Z). CW is funded by Wellcome 222075/Z/20/Z. The views expressed in this manuscript are our own and not an official position of the institution or funder.

Authors' contributions: Study conceptualization and design **–** AEG, CW, APr, CJL, NKN, PvDP, TT. Data analyses – NKN, CJL. First draft of manuscript- NKN, TEM. Laboratory assays provider: APu. Critical review and revision of subsequent drafts and approval of vinal version: all authors.

References

1. UNICEF, UNAIDS. UNAIDS 2021 Estimates: Eliminating mother-to-child transmission. https://data.unicef.org/topic/hivaids/emtct/#more--1656 , *accessed 17 January 2021.*: UNICEF, 2021.
2. Goga A, Chirinda W, Ngandu N, et al. Closing the gaps to eliminate mother-to-child transmission of HIV (MTCT) in South Africa: Understanding MTCT case rates, factors that hinder the monitoring and attainment of targets, and potential game changers. SAMJ 2018;**108**(3 Suppl 1):S17-S24
3. Goga AE, Van de Perre P, Ngandu N, et al. Eliminating HIV transmission through breast milk from women taking antiretroviral drugs. Bmj 2021;**374**:n1697 doi: 10.1136/bmj.n1697[published Online First: Epub Date]|.
4. Goga A, Lombard C, Jackson D, et al. Impact of breastfeeding, maternal antiretroviral treatment and health service factors on 18-month vertical transmission of HIV and HIV-free survival: Results from a nationally representative HIV-exposed infant cohort, South Africa. Journal of Epidemiology and Community Health 2020;**0**:1-9
5. Technau K-G, Kalk E, Coovadia A, et al. Timing of maternal HIV testing and uptake of Prevention of Mother-to-Child Transmission interventions among women and their infected infants in Johannesburg, South Africa. Journal of Acquired Immune Deficiency Syndrome 2014;**65**(5):e170-e78

For peer review only - http://bmjopen.bmj.com/site/about/guidelines.xhtml

6. Myer L, Phillips TK, McIntyre JA, et al. HIV viraemia and mother-to-child transmission risk after antiretroviral therapy initiation in pregnancy in Cape Town, South Africa. HIV Med 2017;**18**(2):80-88 doi: 10.1111/hiv.12397[published Online First: Epub Date]|.
7. Glass TR, Motaboli L, Nsakala B, et al. The viral load monitoring cascade in a resource-limited setting: A prospective multicentre cohort study after introduction of routine viral load monitoring in rural Lesotho. PLoS One 2019;**14**(8):e0220337 doi: 10.1371/journal.pone.0220337[published Online First: Epub Date]|.
8. Ngarina M, Kilewo C, Karlsson K, et al. Virologic and immunologic failure, drug resistance and mortality during the first 24 months postpartum among HIV-infected women initiated on antiretroviral therapy for life in the Mitra plus Study, Dar es Salaam, Tanzania. BMC Infect Dis 2015;**15**:175 doi: 10.1186/s12879-015-0914-z[published Online First: Epub Date]|.
9. Nyakura J, Shewade HD, Ade S, et al. Viral load testing among women on 'option B+' in Mazowe, Zimbabwe: How well are we doing? PLoS One 2019;**14**(12):e0225476 doi: 10.1371/journal.pone.0225476[published Online First: Epub Date]|.
10. Erlwanger AS, Joseph J, Gotora T, et al. Patterns of HIV Care Clinic Attendance and Adherence to Antiretroviral Therapy Among Pregnant and Breastfeeding Women Living With HIV in the Context of Option B+ in Zimbabwe. J Acquir Immune Defic Syndr 2017;**75 Suppl 2**:S198-S206 doi: 10.1097/QAI.0000000000001347[published Online First: Epub Date]|.
11. Van de Perre P, Goga A, Ngandu N, et al. Eliminating postnatal HIV transmission in high incidence areas: need for complementary biomedical interventions. Lancet 2021;**397**(10281):1316-24 doi: 10.1016/S0140-6736(21)00570-5[published Online First: Epub Date]|.
12. Waitt C, Low N, Van de Perre P, et al. Does U=U for breastfeeding mothers and infants? Breastfeeding by mothers on effective treatment for HIV infection in high-income settings. The lancet HIV 2018;**5**:e531–36
13. Woldesenbet SA, Kufa T, Lombard C, et al. The 2017 National Antenatal Sentinel HIV Survey, South Africa: National Department of Health South Africa, National Institute of Communicable Diseases, 2019.
14. World Health Organization. Global guidance on processes and criteria for validation: Elimination of mother-to-child transmission of HIV and syphilis: second edition. 2017. https://apps.who.int/iris/bitstream/handle/10665/259517/9789241513272-eng.pdf: Geneva, 2017.
15. UNAIDS. 90-90-90. An ambitious treatment target to help end the AIDS epidemic. http://files.unaids.org/en/media/unaids/contentassets/documents/unaidspublication/2014/90-90-90_en.pdf: United Nations AIDS, 2014.
16. UNAIDS. FAST-TRACK. Ending the AIDS epidemic by 2030. https://www.unaids.org/sites/default/files/media_asset/JC2686_WAD2014report_en.pdf: United Nations AIDS, 2014.
17. Massyn N, Peer N, Padarath A, et al. District Health Barometer 2015/16. http://www.hst.org.za Health Systems Trust, 2016.
18. Clouse K, Schwartz S, Van Rie A, et al. "What they wanted was to give birth; nothing else": barriers to retention in option B+ HIV care among postpartum women in South Africa. J Acquir Immune Defic Syndr 2014;**67**(1):e12-8 doi: 10.1097/QAI.0000000000000263[published Online First: Epub Date]|.
19. Hosseinipour M, Nelson JAE, Trapence C, et al. Viral Suppression and HIV Drug Resistance at 6 Months Among Women in Malawi's Option B+ Program: Results From the PURE Malawi Study. J Acquir Immune Defic Syndr 2017;**75 Suppl 2**:S149-S55 doi: 10.1097/QAI.0000000000001368[published Online First: Epub Date]|.

For peer review only - http://bmjopen.bmj.com/site/about/guidelines.xhtml

20. Kendall C, Claessens L, Dorward J, et al. Reasons for failure of prevention of mother-to-child HIV transmission in a rural South African district hospital Southern African Journal of HIV Medicine 2015;**16**(1)
21. Myer L, Dunning L, Lesosky M, et al. Frequency of Viremic Episodes in HIV-Infected Women Initiating Antiretroviral Therapy During Pregnancy: A Cohort Study. Clinical infectious diseases : an official publication of the Infectious Diseases Society of America 2017;**64**(4):422-27 doi: 10.1093/cid/ciw792[published Online First: Epub Date]|.
22. South Africa National Department of Health. NATIONAL CONSOLIDATED GUIDELINES for the Prevention of Mother-to-Child Transmission of HIV (PMTCT) and the management of HIV in children, adolescents and adults. https://www.childrenandaids.org/sites/default/files/2017-04/SouthAfrica_National-Consolidated-HIV_2015.pdf: National Department of Health, South Africa, 2015.
23. Harris PA, Taylor R, Thielke R, et al. Research electronic data capture (REDCap)--a metadata-driven methodology and workflow process for providing translational research informatics support. Journal of biomedical informatics 2009;**42**(2):377-81 doi: 10.1016/j.jbi.2008.08.010[published Online First: Epub Date]|.
24. Woldesenbet SA, Kufa T, Barron P, et al. Viral suppression and factors associated with failure to achieve viral suppression among pregnant women in South Africa. AIDS (London, England) 2020;**34**(4):589-97 doi: 10.1097/QAD.0000000000002457[published Online First: Epub Date]|.
25. Landes M, van Lettow M, Nkhoma E, et al. Low detectable postpartum viral load is associated with HIV transmission in Malawi's prevention of mother-to-child transmission programme. J Int AIDS Soc 2019;**22**(6):e25290 doi: 10.1002/jia2.25290[published Online First: Epub Date]|.
26. Siedner MJ, Moorhouse MA, Simmons B, et al. Reduced efficacy of HIV-1 integrase inhibitors in patients with drug resistance mutations in reverse transcriptase. Nature communications 2020;**11**(1):5922 doi: 10.1038/s41467-020-19801-x[published Online First: Epub Date]|.
27. Kintu K, Malaba TR, Nakibuka J, et al. Dolutegravir versus efavirenz in women starting HIV therapy in late pregnancy (DolPHIN-2): an open-label, randomised controlled trial. The lancet HIV 2020;**7**(5):e332-e39 doi: 10.1016/S2352-3018(20)30050-3[published Online First: Epub Date]|.
28. Etta EM, Mavhandu L, Manhaeve C, et al. High level of HIV-1 drug resistance mutations in patients with unsuppressed viral loads in rural northern South Africa. AIDS research and therapy 2017;**14**(1):36 doi: 10.1186/s12981-017-0161-z[published Online First: Epub Date]|.
29. South African National Department of Health. Guideline for the Prevention of Mother to Child Transmission of Communicable Infections. https://www.knowledgehub.org.za/elibrary/guideline-prevention-mother-child-transmission-communicable-infections: National Department of Health, South Africa, October 2019.
30. Phillips TK, Wilson IB, Brittain K, et al. Decreases in Self-Reported ART Adherence Predict HIV Viremia Among Pregnant and Postpartum South African Women. J Acquir Immune Defic Syndr 2019;**80**(3):247-54 doi: 10.1097/QAI.0000000000001909[published Online First: Epub Date]|.
31. Bvochora T, Satyanarayana S, Takarinda KC, et al. Enhanced adherence counselling and viral load suppression in HIV seropositive patients with an initial high viral load in Harare, Zimbabwe: Operational issues. PLoS One 2019;**14**(2):e0211326 doi: 10.1371/journal.pone.0211326[published Online First: Epub Date]|.
32. Shiferaw MB, Yismaw G. Magnitude of delayed turnaround time of laboratory results in Amhara Public Health Institute, Bahir Dar, Ethiopia. Clinical microbiology reviews 2019;**19**(1):240 doi: 10.1128/cmr.00097-18

10.1186/s12913-019-4077-2[published Online First: Epub Date]|.

For peer review only - http://bmjopen.bmj.com/site/about/guidelines.xhtml

33. Fox MP, Brennan AT, Nattey C, et al. Delays in repeat HIV viral load testing for those with elevated viral loads: a national perspective from South Africa. J Int AIDS Soc 2020;**23**(7):e25542 doi: 10.1002/jia2.25542[published Online First: Epub Date]|.
34. Kroidl A, Burger T, Urio A, et al. High turnaround times and low viral resuppression rates after reinforced adherence counselling following a confirmed virological failure diagnostic algorithm in HIV-infected patients on first-line antiretroviral therapy from Tanzania. Tropical medicine & international health : TM & IH 2020 doi: 10.1111/tmi.13373[published Online First: Epub Date]|.
35. Tenthani L, Haas AD, Tweya H, et al. Retention in care under universal antiretroviral therapy for HIV-infected pregnant and breastfeeding women ('Option B+') in Malawi. AIDS (London, England) 2014;**28**(4):589-98 doi: 10.1097/QAD.0000000000000143[published Online First: Epub Date]|.
36. Ngandu NK, Nsibande DF, Magasana V, et al. Quality and turnaround times of PMTCT viral load monitoring under Option B+ in six South African districts with high antenatal HIV burden. South African Medical Journal 2021;**111**(8):759-67 doi: https://doi.org/10.7196/SAMJ.2021.v111i8.15496[published Online First: Epub Date]|.
37. Chugudu K, Kirsten T, Gorogodo B, et al. Demonstration of usability of a digital intervention (DHI) for strengthening the clinic: lab interface for HIV Viral Load testing in a South African clinical setting (Elabs). 9th South African AIDS Conference. Durban South Africa, 2019.
38. Peter J, Barron P, Pillay Y. Using mobile technology to improve maternal, child and youth health and treatment of HIV patients. South African Medical Journal 2016;**106**(1) doi: 10.7196/SAMJ.2016.v106i1.10209[published Online First: Epub Date]|.
39. Lesosky M, Glass T, Mukonda E, et al. Optimal timing of viral load monitoring during pregnancy to predict viraemia at delivery in HIV-infected women initiating ART in South Africa: a simulation study. J Int AIDS Soc 2017;**20 Suppl 7** doi: 10.1002/jia2.25000[published Online First: Epub Date]|.
40. Poulet E, Wolters L, Wapling J, et al. Point-of-Care HIV Viral Load Testing: an Essential Tool for a Sustainable Global HIV/AIDS Response. J Int AIDS Soc 2019;**32**(3) doi: 10.1002/jia2.25387

10.1128/cmr.00097-18[published Online First: Epub Date]|.
41. World Health Organization. Updated recommendations on HIV prevention, Infant diagnosis, Antiretroviral initiation and Monitoring. https://apps.who.int/iris/bitstream/handle/10665/340190/9789240022232-eng.pdf?sequence=1&isAllowed=y, 2021.
42. Ramraj T, Jackson D, Dinh TH, et al. Adolescent Access to Care and Risk of Early Mother-to-Child HIV Transmission. The Journal of adolescent health : official publication of the Society for Adolescent Medicine 2018;**62**(4):434-43 doi: 10.1016/j.jadohealth.2017.10.007[published Online First: Epub Date]|.
43. Fatti G, Shaikh N, Eley B, et al. Adolescent and young pregnant women at increased risk of mother-to-child transmission of HIV and poorer maternal and infant health outcomes: A cohort study at public facilities in the Nelson Mandela Bay Metropolitan district, Eastern Cape, South Africa. South African medical journal = Suid-Afrikaanse tydskrif vir geneeskunde 2014;**104**(12):874-80 doi: 10.7196/samj.8207[published Online First: Epub Date]|.
44. Moyo F, Mazanderani AH, Murray T, et al. Achieving maternal viral load suppression for elimination of mother-to-child transmission of HIV in South Africa. AIDS (London, England) 2021;**35**(2):307-16 doi: 10.1097/QAD.0000000000002733[published Online First: Epub Date]|.
45. Vrazo AC, Firth J, Amzel A, et al. Interventions to significantly improve service uptake and retention of HIV-positive pregnant women and HIV-exposed infants along the prevention of mother-to-child transmission continuum of care: systematic review. Tropical medicine & international health : TM & IH 2018;**23**(2):136-48 doi: 10.1111/tmi.13014[published Online First: Epub Date]|.

For peer review only - http://bmjopen.bmj.com/site/about/guidelines.xhtml

46. Hampanda KM. Intimate partner violence and HIV-positive women's non-adherence to antiretroviral medication for the purpose of prevention of mother-to-child transmission in Lusaka, Zambia. Social science & medicine 2016;**153**:123-30 doi: 10.1016/j.socscimed.2016.02.011[published Online First: Epub Date]|.
47. Hodgson I, Plummer ML, Konopka SN, et al. A systematic review of individual and contextual factors affecting ART initiation, adherence, and retention for HIV-infected pregnant and postpartum women. PLoS One 2014;**9**(11):e111421 doi: 10.1371/journal.pone.0111421[published Online First: Epub Date]|.
48. Ashaba S, Kaida A, Coleman JN, et al. Psychosocial challenges facing women living with HIV during the perinatal period in rural Uganda. PLoS One 2017;**12**(5):e0176256 doi: 10.1371/journal.pone.0176256[published Online First: Epub Date]|.
49. Muchedzi A, Chandisarewa W, Keatinge J, et al. Factors associated with access to HIV care and treatment in a prevention of mother to child transmission programme in urban Zimbabwe. J Int AIDS Soc 2010;**13**:38 doi: 10.1186/1758-2652-13-38[published Online First: Epub Date]|.
50. Ramlagan S, Peltzer K, Ruiter RAC, et al. Prevalence and Factors Associated with Fixed-Dose Combination Antiretroviral Drugs Adherence among HIV-Positive Pregnant Women on Option B Treatment in Mpumalanga Province, South Africa. International journal of environmental research and public health 2018;**15**(1) doi: 10.3390/ijerph15010161[published Online First: Epub Date]|.
51. Maurice Bucagu JdDB, John Muganda, Claire Perrine Humblet. Socio-economic, clinical and biological risk factors for mother - to - child transmission of HIV-1 in Muhima health centre (Rwanda): a prospective cohort study. Archives of Public Health 2013;**71**(4)
52. Hopkins KL, Laher F, Otwombe K, et al. Predictors of HVTN 503 MRK-AD5 HIV-1 gag/pol/nef vaccine induced immune responses. PLoS One 2014;**9**(8):e103446 doi: 10.1371/journal.pone.0103446[published Online First: Epub Date]|.
53. Li X, Ding H, Geng W, et al. Predictive effects of body mass index on immune reconstitution among HIV-infected HAART users in China. BMC Infect Dis 2019;**19**(1):373 doi: 10.1186/s12879-019-3991-6[published Online First: Epub Date]|.
54. Blashill AJ, Mayer KH, Crane HM, et al. Body mass index, immune status, and virological control in HIV-infected men who have sex with men. Journal of the International Association of Providers of AIDS Care 2013;**12**(5):319-24 doi: 10.1177/2325957413488182[published Online First: Epub Date]|.
55. Koethe JR, Jenkins CA, Lau B, et al. Body mass index and early CD4 T-cell recovery among adults initiating antiretroviral therapy in North America, 1998-2010. HIV Med 2015;**16**(9):572-7 doi: 10.1111/hiv.12259[published Online First: Epub Date]|.
56. Bares SH, Smeaton LM, Xu A, et al. HIV-Infected Women Gain More Weight than HIV-Infected Men Following the Initiation of Antiretroviral Therapy. Journal of women's health 2018;**27**(9):1162-69 doi: 10.1089/jwh.2017.6717[published Online First: Epub Date]|.
57. Cois A, Day C. Obesity trends and risk factors in the South African adult population. BMC obesity 2015;**2**:42 doi: 10.1186/s40608-015-0072-2[published Online First: Epub Date]|.
58. The GBD 2015 Obesity Collaborators. Health Effects of overweight and Obesity in 195 ountries over 25 Years. The New England Journal of Medicine 2017;**377**(1)

Supplementary files

Supplementary File 1

For peer review only - http://bmjopen.bmj.com/site/about/guidelines.xhtml

Title: STROBE 2007 (v4) Statement—Checklist of items that should be included in reports of *cross-sectional studies*
Format: pdf, table

Supplementary File 2

Title: Summary of participant characteristics by maternal viral load non-suppression per study group.

Format: pdf, table

For peer review only

For peer review only - http://bmjopen.bmj.com/site/about/guidelines.xhtml

STROBE 2007 (v4) Statement—Checklist of items that should be included in reports of *cross-sectional studies*

**Section/Topic**	**Item #**	**Recommendation**	**Reported on page #**
**Title and abstract**	1	(*a*) Indicate the study's design with a commonly used term in the title or the abstract	1
		(*b*) Provide in the abstract an informative and balanced summary of what was done and what was found	2
**Introduction**			
Background/rationale	2	Explain the scientific background and rationale for the investigation being reported	4
Objectives	3	State specific objectives, including any prespecified hypotheses	5
**Methods**			
Study design	4	Present key elements of study design early in the paper	5
Setting	5	Describe the setting, locations, and relevant dates, including periods of recruitment, exposure, follow-up, and data collection	5
Participants	6	(*a*) Give the eligibility criteria, and the sources and methods of selection of participants	5
Variables	7	Clearly define all outcomes, exposures, predictors, potential confounders, and effect modifiers. Give diagnostic criteria, if applicable	6, 7
Data sources/ measurement	8*	For each variable of interest, give sources of data and details of methods of assessment (measurement). Describe comparability of assessment methods if there is more than one group	6, 7
Bias	9	Describe any efforts to address potential sources of bias	7
Study size	10	Explain how the study size was arrived at	5
Quantitative variables	11	Explain how quantitative variables were handled in the analyses. If applicable, describe which groupings were chosen and why	6, 7
Statistical methods	12	(*a*) Describe all statistical methods, including those used to control for confounding	7
		(*b*) Describe any methods used to examine subgroups and interactions	
		(*c*) Explain how missing data were addressed	6,7
		(*d*) If applicable, describe analytical methods taking account of sampling strategy	7
		(*e*) Describe any sensitivity analyses	
**Results**			

For peer review only

For peer review only - http://bmjopen.bmj.com/site/about/guidelines.xhtml

Participants	13*	(a) Report numbers of individuals at each stage of study—eg numbers potentially eligible, examined for eligibility, confirmed eligible, included in the study, completing follow-up, and analysed	7
		(b) Give reasons for non-participation at each stage	
		(c) Consider use of a flow diagram	
Descriptive data	14*	(a) Give characteristics of study participants (eg demographic, clinical, social) and information on exposures and potential confounders	7-10
		(b) Indicate number of participants with missing data for each variable of interest	
Outcome data	15*	Report numbers of outcome events or summary measures	
Main results	16	(*a*) Give unadjusted estimates and, if applicable, confounder-adjusted estimates and their precision (eg, 95% confidence interval). Make clear which confounders were adjusted for and why they were included	11-16
		(*b*) Report category boundaries when continuous variables were categorized	
		(*c*) If relevant, consider translating estimates of relative risk into absolute risk for a meaningful time period	
Other analyses	17	Report other analyses done—eg analyses of subgroups and interactions, and sensitivity analyses	
**Discussion**			
Key results	18	Summarise key results with reference to study objectives	17
Limitations	19	Discuss limitations of the study, taking into account sources of potential bias or imprecision. Discuss both direction and magnitude of any potential bias	3, 20
Interpretation	20	Give a cautious overall interpretation of results considering objectives, limitations, multiplicity of analyses, results from similar studies, and other relevant evidence	20
Generalisability	21	Discuss the generalisability (external validity) of the study results	20
**Other information**			
Funding	22	Give the source of funding and the role of the funders for the present study and, if applicable, for the original study on which the present article is based	21

*Give information separately for cases and controls in case-control studies and, if applicable, for exposed and unexposed groups in cohort and cross-sectional studies.

Note: An Explanation and Elaboration article discusses each checklist item and gives methodological background and published examples of transparent reporting. The STROBE checklist is best used in conjunction with this article (freely available on the Web sites of PLoS Medicine at http://www.plosmedicine.org/, Annals of Internal Medicine at http://www.annals.org/, and Epidemiology at http://www.epidem.com/). Information on the STROBE Initiative is available at www.strobe-statement.org.

For peer review only

Summary of participant characteristics by maternal viral load non-suppression per study group

	Third trimester		0-14 weeks postpartum		15-26 weeks postpartum		27-52 weeks postpartum		53-104 weeks postpartum	
	N	% VL>1000 [95% CI]	N	% VL>1000 [95% CI]	N	% VL>1000 [95% CI]	N	% VL>1000 [95% CI]	N	% VL>1000 [95% CI]
All	176	17.1 [11.7, 24.2]	128	15.0 [8.5, 25.2]	75	6.9 [2.2, 20.1]	123	17.5 [9.0, 31.2]	110	16.4 [9.3, 27.2]
**Socio-demographics and antenatal care**										
Age in years										
15–24	33	28.7 [13.2, 51.6]	23	3.1 [0.2, 28.9]	12	16.9 [1.2, 76.6]	17	28.0 [10.4, 56.5]	20	39.0 [25.9, 54.0]
25-34	99	15.4 [7.7, 28.4]	75	17.0 [6.8, 36.4]	48	6.0 [1.5, 21.5]	73	16.7 [6.3, 37.4]	56	14.5 [4.4, 38.3]
35-46	44	12.1 [3.2, 36.8]	30	20.2 [7.3, 44.9]	15	0.0	33	14.2 [3.9, 40.8]	34	5.3 [1.2, 20.6]
P-values		*0.332*		*0.288*		*0.471*		*0.538*		***0.016***
BMI										
13.0 to 24.9	33	12.1 [3.2, 36.4]	48	19.1 [5.7, 47.8]	33	12.7 [4.2, 32.6]	53	12.8 [6.2, 24.4]	53	19.0 [8.1, 38.6]
25 to 29.9	55	25.9 [15.1, 40.7]	41	20.8 [6.4, 50.3]	19	4.3 [0.4, 33.2]	39	25.2 [7.0, 60.3]	21	23.3 [12.2, 39.9]
30 to 76.7	86	13.9 [7.9, 23.2]	39	2.1 [0.2, 19.3]	22	0.0	30	16.7 [7.5, 33.2]	35	9.0 [3.4, 21.4]
P-values		*0.175*		*0.274*		*0.115*		*0.351*		*0.184*
Education										
>12 years of education	59	17.9 [6.3, 41.6]	59	13.1 [6.7, 24.0]	23	3.9 [0.6, 21.3]	43	22.1 [9.4, 43.7]	29	8.6 [1.3, 40.3]
≤12 years of education	117	16.7 [9.7, 27.2]	69	16.4 [6.9, 34.1]	52	8.1 [2.4, 24.0]	80	14.9 [8.8, 23.9]	81	19.3 [10.6, 32.4]
P-values		*0.899*		*0.644*		*0.335*		*0.192*		*0.324*
Married/Cohabiting										
No	114	15.3 [8.0, 27.3]	77	8.2 [4.0, 16.2]	46	1.9 [0.2, 17.2]	68	18.5 [11.1, 29.2]	57	16.1 [7.8, 30.2]

For peer review only - http://bmjopen.bmj.com/site/about/guidelines.xhtml

Yes	62	20.7 [11.1, 35.2]	51	24.5 [9.5, 49.9]	29	13.1 [4.7, 31.6]	55	16.4 [3.8, 49.0]	53	16.6 [7.9, 31.6]
P-values		*0.480*		*0.083*		***0.045***		*0.846*		*0.929*
Income source[a]										
Employed	47	13.4 [6.1, 27.1]	41	8.6 [2.3, 27.4]	22	0.0	26	11.6 [2.7, 37.9]	27	22.0 [5.0, 60.3]
Spouse/partner	54	20.5 [9.3, 39.1]	37	24.3 [6.3, 60.5]	19	19.0 [6.5, 44.0]	40	18.8 [4.5, 53.5]	37	15.4 [6.9, 30.6]
Parent/relative	34	24.2 [7.0, 57.4]	17	30.1 [9.5, 63.8]	9	10.3 [0.6, 69.5]	14	24.4 [8.1, 54.2]	16	26.7 [6.8, 64.5]
Grant	38	12.7 [3.9, 33.8]	32	4.7 [0.9, 20.2]	24	0.0	43	17.3 [7.0, 36.8]	29	9.0 [1.7, 35.9]
P-values		*0.580*		*0.135*		*0.146*		*0.754*		*0.513*
Household gross income/month										
>R3200	72	21.7 [15.4, 29.7]	60	15.1 [6.0, 33.1]	34	5.2 [1.4, 18.0]	55	27.2 [14.6, 45.0]	41	18.7 [6.3, 43.9]
R3200 or less/none	104	13.4 [6.8, 24.9]	68	14.9 [7.6, 27.2]	41	8.1 [1.5, 33.1]	68	9.8 [5.6, 16.4]	68	15.5 [8.3, 27.0]
P-values		*0.168*		*0.983*		*0.642*		***0.007***		*0.711*
Partner's HIV status[a]										
Negative	30	24.1 [13.1, 40.1]	17	3.7 [0.2, 37.5]	15	0.0	28	11.4 [4.4, 26.5]	20	5.1 [0.4, 41.2]
Positive	71	11.8 [3.3, 34.2]	53	18.1 [7.8, 36.8]	38	14.7 [5.1, 35.5]	60	17.7 [9.3, 31.0]	57	12.5 [3.9, 33.1]
Don't know	75	19.2 [10.3, 32.9]	57	16.4 [6.3, 36.5]	22	0.0	35	21.2 [7.7, 46.6]	33	27.3 [20.2, 35.7]
P-values		*0.429*		*0.354*		***0.035***		*0.429*		*0.137*
Condom use frequency[a]										
Never	26	23.5 [12.8, 39.2]	11	11.8 [2.0, 46.4]	1	0.0	7	0.0	7	18.2 [4.2, 53.1]
Sometimes	72	17.4 [9.0, 30.9]	46	14.1 [2.4, 52.3]	24	3.1 [0.3, 23.2]	39	15.7 [8.3, 27.9]	45	6.1 [2.3, 15.3]

For peer review only - http://bmjopen.bmj.com/site/about/guidelines.xhtml

Always	78	15.0 [7.0, 29.4]	70	16.5 [8.1, 30.7]	49	9.3 [2.6, 28.4	75	19.7 [7.7, 42.0]	58	22.3 [13.9, 33.8]
P-values		*0.560*		*0.920*		*0.582*		*0.501*		***0.006***
Planned pregnancy										
No	99	13.1 [6.2, 25.6]	75	14.3 [4.6, 36.6]	44	8.8 [2.2, 28.8]	58	19.9 [11.6, 31.9]	54	13.5 [5.7, 28.5]
Yes	77	22.0 [14.0, 32.9]	53	16.0 [6.9, 32.9]	31	4.3 [0.7, 21.0]	65	15.3 [4.1, 43.6]	56	18.6 [9.3, 33.5]
P-values		*0.210*		*0.871*		*0.444*		*0.646*		*0.479*
Gestation at ANC-1 visit										
≤12 weeks	75	12.7 [4.6, 30.4]	86	13.1 [4.7, 31.6]	56	6.4 [1.5, 23.2]	84	19.1 [9.2, 35.5]	78	15.2 [8.0, 26.8]
13-20 weeks	66	21.1 [13.8, 30.9]	33	12.7 [3.2, 38.8]	11	13.8 [2.2, 53.6]	29	8.3 [1.6, 32.6]	26	24.8 [12.9, 42.5]
>20 weeks	35	19.7 [8.6, 39.0]	9	34.3 [15.1,60.4]	8	0.0	10	29.9 [5.2, 76.9]	6	0.0 [0.0, 0.0]
P-values		*0.442*		*0.276*		*0.418*		*0.362*		*0.201*
Number of ANC visits[a]										
0-4 visits	93	16.7 [8.3, 30.8]	34	20.9 [9.3, 40.5]	17	4.6 [0.5, 31.7]	33	14.9 [4.6, 38.6]	33	14.1 [6.9, 26.6]
5-12 visits	83	17.5 [12.6, 23.7]	93	12.8 [7.2, 21.6]	58	7.6 [2.1, 23.7]	90	18.5 [9.4, 33.2]	77	17.5 [8.8, 31.6]
P-values		*0.893*		*0.139*		*0.646*		*0.653*		*0.550*
**Primary exposure variables**										
Timing of HIV-positive result										
Before pregnancy	128	14.2 [7.4, 25.6]	81	11.1 [5.6, 20.8]	59	7.8 [2.6, 21.5]	95	15.6 [5.9, 35.2]	85	14.0 [6.3, 28.4]
At ANC-1 or after	48	24.8 [17.2, 34.3]	47	22.0 [7.0, 51.3]	16	4.3 [0.3, 43.4]	28	23.8 [8.3, 52.1]	25	23.7 [11.9, 41.5]
P-values		*0.108*		*0.296*		*0.607*		*0.523*		*0.231*
Time since ART initiation										
>12 months	97	9.8	75	9.6	61	7.5	102	15.9	107	15.9

For peer review only - http://bmjopen.bmj.com/site/about/guidelines.xhtml

		[5.5, 16.8]		[5.5, 16.2]		[2.4, 21.0]		[6.5, 33.9]		[8.7, 27.4]
≤12 months	79	25.8 [19.9, 32.7]	53	22.7 [9.7, 44.6]	14	4.9 [0.3, 47.6]	*21*	24.8 [6.7, 60.2]	3	34.2 [8.5, 74.3]
P-values		***0.001***		*0.093*		*0.715*		*0.539*		*0.196*
Current ART regimen[a]										
2nd/3rd line or unknown	28	21.2 [9.3, 41.5]	29	5.4 [1.1, 22.7]	14	4.8 [0.4, 36.1]	26	3.4 [0.2, 37.3]	22	8.6 [1.9, 31.2]
First line	147	16.5 [11.0, 24.0]	99	17.6 [9.9, 29.2]	61	7.5 [1.9, 25.6]	97	21.6 [12.6, 34.3]	87	18.3 [10.1, 30.8]
P-values		*0.479*		*0.136*		*0.717*		***0.048***		*0.251*
Missed an ART dose last 7 days										
No	168	16.7 [11.3, 24.0]	123	12.2 [8.0, 18.0]	72	7.2 [2.3, 20.5]	116	18.6 [9.8, 32.4]	103	16.3 [9.0, 27.5]
Yes	8	26.3 [5.3, 69.3]	5	67.1 [8.3, 97.9]	3	0.0	7	0.0	7	18.2 [4.2, 53.1]
P-values		*0.455*		***0.029***		*0.545*		*0.066*		*0.850*
Facing any ART adherence challenges										
No	121	12.6 [7.2, 21.2]	60	17.3 [7.9, 33.7]	43	6.4 [0.9, 33.5]	72	14.7 [8.1, 25.1]	80	16.7 [8.3, 30.6]
Yes	55	25.0 [13.9, 40.8]	68	12.2 [5.4, 25.1]	32	7.7 [1.9, 26.9]	51	21.1 [5.3, 56.1]	30	15.5 [4.6, 41.1]
P-values		*0.078*		*0.503*		*0.863*		*0.616*		*0.903*
**Infant related factors** (postpartum **sample only)**										
All			*127*	*15.0 [8.5, 25.2]*	*75*	*6.9 [2.2, 20.1]*	*123*	*17.5 [9.0, 31.2]*	*110*	*16.4 [9.3, 27.2]*
Infant ever breastfed										
No			49	25.1 [8.7, 54.1]	21	3.0 [0.4, 21.4]	42	22.2 [9.3, 44.1]	34	14.3 [5.4, 32.7]
Yes			79	8.3 [3.7, 17.6]	53	8.3 [2.4, 25.3]	81	15.3 [7.9, 27.6]	76	17.2 [9.0, 30.3]
P-values				*0.138*		*0.355*		*0.268*		*0.668*
Infant currently breastfeeding										

For peer review only - http://bmjopen.bmj.com/site/about/guidelines.xhtml

Yes			60	9.4 [3.7, 21.8]	35	10.2 [3.2, 28.1]	48	14.4 [5.7, 32.0]	20	19.6 [9.3, 36.7]
No			19	5.3 [0.7, 32.1]	18	4.3 [0.3, 39.7]	33	16.5 [9.4, 27.4]	56	16.3 [6.9, 33.8]
No response			49	25.1 [8.7, 54.1]	22	2.8 [0.3, 20.9]	42	22.2 [9.3, 44.1]	34	14.3 [5.4, 32.7]
P-values				*0.169*		*0.398*		*0.401*		*0.797*
Infant currently on ARV	prophylaxis [a]									
No			37	15.2 [6.3, 32.5]	53	4.1 [1.2, 13.3]	104	15.0 [8.0, 26.2]	99	16.2 [8.2, 29.4]
Yes			90	15.1 [8.1, 26.5]	22	12.8 [3.1, 40.4]	18	26.1 [8.5, 57.5]	10	23.6 [4.0, 69.4]
P-values				*0.984*		*0.161*		*0.145*		*0.627*
Infant HIV status at enrolment										
Negative			74	17.5 [8.9, 31.6]	61	8.6 [2.7, 24.3]	113	19.0 [10.1, 33.0]	97	13.3 [6.3, 25.9]
Positive			3	0.0	1	0.0	1	0.0	5	23.1 [3.0, 74.7]
Unknown			51	12.2 [5.8, 24.1]	13	0.0	9	0.0	8	37.3 [15.9, 65.3]
P-values				*0.473*		*0.564*		*0.330*		*0.930*
Gestational age at birth[a]										
≤37 weeks			17	20.6 [12.0, 33.1]	7	0.0	20	3.0 [0.3, 27.8]	23	31.1 [21.4, 42.8]
38 - 42 weeks			110	11.9 [7.3, 19.0]	67	8.4 [2.4, 25.5]	103	20.3 [10.2, 36.3]	87	10.9 [3.9, 26.8]
P-values				*0.154*		*0.510*		*0.078*		*0.086*
Infant birth weight[a]										
Birth weight≥2.5kg			116	14.0 [7.2, 25.4]	71	6.4 [1.7, 21.0]	109	16.9 [10.3, 26.4]	96	15.8 [8.8, 26.9]
Low birth weight			12	26.2 [17.1,38.0]	3	16.7 [0.9, 81.9]	14	22.5 [3.7, 68.9]	14	20.3 [4.8, 56.0]
P-values				*0.062*		*0.499*		*0.59*		*0.68*

For peer review only - http://bmjopen.bmj.com/site/about/guidelines.xhtml

[a]denominator less than N due to missing responses; BMI – body mass index; ART antiretroviral therapy; ARV- antiretroviral; PMTCT – prevention of mother-to-child transmission of HIV; ANC – antenatal care; CI – confidence interval. P-values are from a chi-squared test. Significant p-values <0.05 are in boldface font.

For peer review only
